# Supplementary material for: A sleep-active neuron can promote survival while sleep behavior is disturbed
Source: PLoS Genet. 2023 Mar 14;19(3):e1010665. doi: 10.1371/journal.pgen.1010665 (PMC10038310; doi:10.1371/journal.pgen.1010665)
Supplement: S1 Text — (DOCX) [file pgen.1010665.s001.docx]

CRISPR sequences

PHX1433: *flp-11(syb1433[flp-11-SL2-egl-23cDNA(A383V)-linker-mKate2])*X

>flp-11b-SL2(gpd-2)-egl-23(n601)-linker(GSGSGSGSG)-mKate2(two introns)

ctattgtagTGCGGAGAAACGTGCCATGCGGAACGCCTTGGTTCGATTTGGAAGAGCTAGTGGTGGAATGAGAAATGCTCTCGTTAGATTCGGAAAGAGGTCTCCATTGGACGAGGAAGACTTTGCTCCAGAGAGCCCACTCCAGGGAAAACGGAACGGTGCCCCACAACCATTTGgtaagttgtcttaaaatttttcttccgctttttgcctttgcttcatgtgtcgtttattttgctttgcagttcgctttggccgatccggtcaactcgaccacatgcacgaccttttgtcgactcttcagAAGCTCAAGTTCGCCAACAACAAGTAATGACCGAGGACGACCGTCTTCTGCTCGAACAACTCCTG**CGA**CGAATTCATCATTAAgctgtctcatcctactttcacctagttaactgcttgtcttaaaatctatgcttctctttagtatctaaaattttcctagaagcttacaagtatataaatggtctcttctcaataaaggttgtatatttattcatcttattgaatctgccatttcctcgtttttgcgagtttatataccttccaattttctttctattgtattttcaacttctaattttaattcagggaaactgcttcaacgcatcATGAAGCTCACGTTGAAGAAATCCGTATTCTCAAGGGATAAACATATCTTGCAAAAGGCGACACCACTATTCGTTCACTTTCTAATGATAGTAAGTGTGGGTGCCTACGCAATATTTGGAGCACTTGTAATGAGAAGTCTTGAATCGAGAACTGTCACAAGTATTGAAAAGAAGACGGATGTTCACAGAAGACATGTTAATTTGACTAATTTTCAACATCCACCAACTCCAATCACATTAGAGCAAAGACATCGACGGAGACGCAGACATAATGAGACAGCTTTGGAAGACCATTTGTCTGAAAAATTATCCAGAGAAAAACGTGCAGCAGCGCATATCATGAGAAGTCGAAAATGCGTTATCAGTGTGATAAAGAAAATGTCAAGCATGGAATGTTCATTTGACACTCTCGACGAGAAGCTCGTAAAAGCACTCGATGAATGTTACCACGTGGCAGTTGAACATAATACTCATGTGAATCATGTACTTTTCACGAATAGTAAGGAAGAAGTGGAGTCAGTTGGGGAAGAAGCAGAAGAAGATGTTTCCGAATGGTCATTTATGGACTCGTTGTTGTTTGCATTCACTGTTATTACGACGATTGGATACGGAAACGTGGCACCTCGAACATTTGGTGGCCGTCTATTTGTCATTGGTTATGGTCTAATTGGTATTCCGTTTACACTGCTGGCAATTGCAGATCTCGGAAAATTCATATCAGAAATGATGGTGGAGGCGAAAAGTTTTTGTAGGAAAACCTGGAAAAAACTCAAAAAAGCGTGGAACCCGAATTTCATTCGCGCAAAGGATCTTTCAAATACGGATATTGAGGAGAAAATATTGGATAATGAGAAAATCGAAAATGAGCCGGAAACTTCAGAAGTATCAGAAGAAGAAGACGATTTGACAGAGACAGAAGCCACGTCACTTTTCATTTTATTTTTGGTTTATATCGCATTTGGAGGGTTCATGTTAGCTGCTTATGAACCTGATATGGACTTTTTCAAAGCGGTCTACTTTAATTTTGTGACATTGACATCAATTGGTCTGGGAGATATTGTACCGAGAAGTGAAACCTATATGCTCATTACAATAGTCTACATTGCAATTGGTCTTGCTCTTACCACAATTGCCATTGAAATCGCC**GTA**GATGCATTGAAGAAGCTGCATTATTTTGGAAGAAAAATCGAAAATGTTGGAAATGTGGCTATATGGTTTGGAGGAAAGAAAATTACAATGAAAGCACTGGTCAAAAACCTCGGTGACCAGTTCAATCTTCCAACTACCGTAGTCAAGAATTTGAATTTGGATCATTTTGTGGATCAAGCGATTAAAGTGGAGGAAGGAGAGATTGAGACACTCAGACCGCCGCCTTATGAGCCACCGTCTGATCGATTTGAAGCTGAATTCGCTGATGAACCAGAATGTGAATGGATCCGTGATCCGACTCCAACTCCACCCCCATCACCTCAACCGGTTTATCGTCTTCCATCTCCAAAACCAGTTACACCGGAACCTCTACCAAGTCCAACAATAACTGATGTATCACTTGCGATTGCAACACCATCACCTGAAGAATCGGATGATGATCAAGAACTAATTCTCCCATCACCTGAACCGAGTCCAGTTCGAGAGCCAACTCCACCACCGCCTCCACGGGAGCCAACACCTCGTGAGCCAACTCCTGAGCCAGAGCCAGTTCGAGAGCCGACGCCTCCACCTCCGCCACCTGCCAAGCCCCGTCCACTGACTGCCGCTGAAATCGCGGCTCAAAAACGCAAAGCGTACAGCGAAGAAGCATGGCGTCGATACCAAGAATACCAGAAACAATGGAAGAAGTTCCGTCAAACTCAGAAAACTCCCGCACCATCTGGAGCCTCTACATCAGGGGCATCAACATCAAAACCGTCTGGAACATCACCGGAAAGTGGAGCCGGAGTATGTACTGGACCATCAACAAGGAGTCAATCAATAACATCAGTTGCATCTGGAAAGACATCAAGAAGTGCAACACCGGAAAGCAAGAAATCATCACATTTGAGTGGCTCATCGAGAAGAGAAAGCGGTGGAAAAGGATCCGGATCCGGATCCGGATCCGGAATGTCCGAGCTCATCAAGGAGAACATGCACATGAAGCTCTACATGGAGGGAACCGTCAACAACCACCACTTCAAGTGCACCTCCGAGGGAGAGGGAAAGCCATACGAGGGAACCCAAACCATGCGTATCAAGgtaagtttaaacatatatatactaactaaccctgattatttaaattttcagGCCGTCGAGGGAGGACCACTCCCATTCGCCTTCGACATCCTCGCCACCTCCTTCATGTACGGATCCAAGACCTTCATCAACCACACCCAAGGAATCCCAGACTTCTTCAAGCAATCCTTCCCAGAGGGATTCACCTGGGAGCGTGTCACCACCTACGAGGACGGAGGAGTCCTCACCGCCACCCAAGACACCTCCCTCCAAGACGGATGCCTCATCTACAACGTCAAGATCCGTGGAGTCAACTTCCCATCCAACGGACCAGTCATGCAAAAGAAGACCCTCGGATGGGAGGCCTCCACCGAGACCCTCTACCCAGCCGACGGAGGACTCGAGGGACGTGCCGACATGGCCCTCAAGCTCGTCGGAGGAGGACACCTCATCTGCAACCTCAAGgtaagtttaaacatgattttactaactaactaatctgatttaaattttcagACCACCTACCGTTCCAAGAAGCCAGCCAAGAACCTCAAGATGCCAGGAGTCTACTACGTCGACCGTCGTCTCGAGCGTATCAAGGAGGCCGACAAGGAGACCTACGTCGAGCAACACGAGGTCGCCGTCGCCCGTTACTGCGACCTCCCATCCAAGCTCGGACACCGTTAAaaatcatatgtttttctctctcacactctcttttttc

PHX1445: *aptf-1(gk794)II; flp-11(syb1445[flp-11-SL2-unc-58(L428F)-linker-mKate2])*X

>flp-11b-SL2(gpd-2)-unc-58(e665)-linker(GSGSGSGSG)-mKate2(two introns)

TGGAAGAGCTAGTGGTGGAATGAGAAATGCTCTCGTTAGATTCGGAAAGAGGTCTCCATTGGACGAGGAAGACTTTGCTCCAGAGAGCCCACTCCAGGGAAAACGGAACGGTGCCCCACAACCATTTGgtaagttgtcttaaaatttttcttccgctttttgcctttgcttcatgtgtcgtttattttgctttgcagttcgctttggccgatccggtcaactcgaccacatgcacgaccttttgtcgactcttcagAAGCTCAAGTTCGCCAACAACAAGTAATGACCGAGGACGACCGTCTTCTGCTCGAACAACTCCTG**CGA**CGAATTCATCATTAAgctgtctcatcctactttcacctagttaactgcttgtcttaaaatctatgcttctctttagtatctaaaattttcctagaagcttacaagtatataaatggtctcttctcaataaaggttgtatatttattcatcttattgaatctgccatttcctcgtttttgcgagtttatataccttccaattttctttctattgtattttcaacttctaattttaattcagggaaactgcttcaacgcatcATGGCTCCACTGACTGTGAAAAGCTCACCTCCAAAAAAGGCAAAAGGAATATCAAAATTTCGGAGAAAAAAGAAGCAGCCACCACCAGACTCAACCGTATTCGTCGCATGGGCACTCCGAAGTGTCCGAGAGTCTCTGATCCAAGTTGATCCATTGGCCGCTGCACTTGCACATCAGGCTCGAAAGACAAATAGTGTGCCAGCTGTCTCGAGAACTCCACTGCTTCTACAGTTCACTCCTTTCGGACCACCTCTCAGTGCGTATCATGTGACAGCTCGGTGGGAAGGTGCAAATATCAATTCACAATCAGCATTGCTCGATGCAGATGATGGAGCTACAGTTATCACAGATACCATCAAAGATGACCAAGATGATAAAGAACCAAAAAGCTGCCCGCAACAGACTGTCAAATACATCAAAATACTTACACCTCACGTGATCTTGGTGTCAGTGTTAATTGGATATTTATGCTTGGGAGCTTGGATACTCATGTTACTGGAAACAAGGACGGAACTTCTTGCCAGATCCAAAAAACTTGTCAGGTTAACAAATTTGATGTCAAACTTCACTGCCGAAAGTTGGAAGATGCTCAATAATGCTCAACACGGGGTTAGTAATATGGATGAAGGTGAATGGGCTGCAACATTTCGAGAATGGATGGTACGAGTATCAGAAACAGTGGACGATAGGAGACCTATACGACGTGAATTAAACCGGCCTGATGACTTATCAAATATGCATAATAAATGGACATTTCCAACTGCAATATTATATGTTCTCACTGTGTTAACTACTTGCGGTTATGGAGAAGTATCTGTCGACACAGACGTCGGAAAGGTTTTCTCAGTAGCATTCGCGCTTGTTGGTATACCACTTATGTTCATAACAGCTGCCGATATTGGTAAATTTTTATCTGAAACATTACTCCAGTTTGTGAGCTTTTGGAATCGAAGTGTCCGAAAAGTGAAGCAATGGATGAGTCGTATTCGTCACGGCAGGAGAAAGTCATTACAATCAACGGGTGGTCCCAACGATACTCTCGATATTCTTGGTGTCGACGGAACTGAAGAGAAACTTTGGTTCCCAATAGGTGCATATGTATCATGTATTTGCATATATTGCTCAATTGGGTCTGCCATGTTTATCACATGGGAAAGAACTTGGTCTTTCATTCATGCGTTTCATTTTGGTTTCAATTTGATTGTAACAGTCGGACTCGGAGATATCGTTGTGACTGATTACATATTTTTATCACTTATCGTTGCATTTGTGATAGTTGGTTTTTCCGTAGTGACCATGTGCGTGGATCTTGCGTCCACACATCTCAAGGCGTACTTCACCAGAATTCACTACTTTGGTCGAGCAAAACGATTCTTAGGAATGAGTGAGGAACTCAAAGAAATCGTTGCTTTACTGGGGGCGATGCGACGGAAAAAAGGCGGTAAAGTTACATGGAATGATGTGCGAGACTTCCTGGATAACGAACTCCGCGATCGACCTTTTGAACCTCATGAGCTTCTGATGAAGCTCAGATTTATTGACGAAACATCTTCTGGAATGTCTACAATCCGTCACAATTCCTTCCAGTCAGATTTTTTCCGAGAATCAGAGTACATCCGAAGAGTGGCTGCGCTGAGGCCAGAACAGCCAGCATATTTGGGATCCGGATCCGGATCCGGATCCGGAATGTCCGAGCTCATCAAGGAGAACATGCACATGAAGCTCTACATGGAGGGAACCGTCAACAACCACCACTTCAAGTGCACCTCCGAGGGAGAGGGAAAGCCATACGAGGGAACCCAAACCATGCGTATCAAGgtaagtttaaacatatatatactaactaaccctgattatttaaattttcagGCCGTCGAGGGAGGACCACTCCCATTCGCCTTCGACATCCTCGCCACCTCCTTCATGTACGGATCCAAGACCTTCATCAACCACACCCAAGGAATCCCAGACTTCTTCAAGCAATCCTTCCCAGAGGGATTCACCTGGGAGCGTGTCACCACCTACGAGGACGGAGGAGTCCTCACCGCCACCCAAGACACCTCCCTCCAAGACGGATGCCTCATCTACAACGTCAAGATCCGTGGAGTCAACTTCCCATCCAACGGACCAGTCATGCAAAAGAAGACCCTCGGATGGGAGGCCTCCACCGAGACCCTCTACCCAGCCGACGGAGGACTCGAGGGACGTGCCGACATGGCCCTCAAGCTCGTCGGAGGAGGACACCTCATCTGCAACCTCAAGgtaagtttaaacatgattttactaactaactaatctgatttaaattttcagACCACCTACCGTTCCAAGAAGCCAGCCAAGAACCTCAAGATGCCAGGAGTCTACTACGTCGACCGTCGTCTCGAGCGTATCAAGGAGGCCGACAAGGAGACCTACGTCGAGCAACACGAGGTCGCCGTCGCCCGTTACTGCGACCTCCCATCCAAGCTCGGACACCGTTAAaaatcatatgtttttctctctcacactctcttttttc

PHX1464: *flp-11(syb1464[flp-11-SL2-egl-23cDNA(L229N)-linker-mKate2])*X

>flp-11b-SL2(gpd-2)-egl-23(L229N)-linker(GSGSGSGSG)-mKate2(two introns)

ctattgtagTGCGGAGAAACGTGCCATGCGGAACGCCTTGGTTCGATTTGGAAGAGCTAGTGGTGGAATGAGAAATGCTCTCGTTAGATTCGGAAAGAGGTCTCCATTGGACGAGGAAGACTTTGCTCCAGAGAGCCCACTCCAGGGAAAACGGAACGGTGCCCCACAACCATTTGgtaagttgtcttaaaatttttcttccgctttttgcctttgcttcatgtgtcgtttattttgctttgcagttcgctttggccgatccggtcaactcgaccacatgcacgaccttttgtcgactcttcagAAGCTCAAGTTCGCCAACAACAAGTAATGACCGAGGACGACCGTCTTCTGCTCGAACAACTCCTG**CGA**CGAATTCATCATTAAgctgtctcatcctactttcacctagttaactgcttgtcttaaaatctatgcttctctttagtatctaaaattttcctagaagcttacaagtatataaatggtctcttctcaataaaggttgtatatttattcatcttattgaatctgccatttcctcgtttttgcgagtttatataccttccaattttctttctattgtattttcaacttctaattttaattcagggaaactgcttcaacgcatcATGAAGCTCACGTTGAAGAAATCCGTATTCTCAAGGGATAAACATATCTTGCAAAAGGCGACACCACTATTCGTTCACTTTCTAATGATAGTAAGTGTGGGTGCCTACGCAATATTTGGAGCACTTGTAATGAGAAGTCTTGAATCGAGAACTGTCACAAGTATTGAAAAGAAGACGGATGTTCACAGAAGACATGTTAATTTGACTAATTTTCAACATCCACCAACTCCAATCACATTAGAGCAAAGACATCGACGGAGACGCAGACATAATGAGACAGCTTTGGAAGACCATTTGTCTGAAAAATTATCCAGAGAAAAACGTGCAGCAGCGCATATCATGAGAAGTCGAAAATGCGTTATCAGTGTGATAAAGAAAATGTCAAGCATGGAATGTTCATTTGACACTCTCGACGAGAAGCTCGTAAAAGCACTCGATGAATGTTACCACGTGGCAGTTGAACATAATACTCATGTGAATCATGTACTTTTCACGAATAGTAAGGAAGAAGTGGAGTCAGTTGGGGAAGAAGCAGAAGAAGATGTTTCCGAATGGTCATTTATGGACTCGTTGTTGTTTGCATTCACTGTTATTACGACGATTGGATACGGAAACGTGGCACCTCGAACATTTGGTGGCCGTCTATTTGTCATTGGTTATGGTCTAATTGGTATTCCGTTTACA**AAC**CTGGCAATTGCAGATCTCGGAAAATTCATATCAGAAATGATGGTGGAGGCGAAAAGTTTTTGTAGGAAAACCTGGAAAAAACTCAAAAAAGCGTGGAACCCGAATTTCATTCGCGCAAAGGATCTTTCAAATACGGATATTGAGGAGAAAATATTGGATAATGAGAAAATCGAAAATGAGCCGGAAACTTCAGAAGTATCAGAAGAAGAAGACGATTTGACAGAGACAGAAGCCACGTCACTTTTCATTTTATTTTTGGTTTATATCGCATTTGGAGGGTTCATGTTAGCTGCTTATGAACCTGATATGGACTTTTTCAAAGCGGTCTACTTTAATTTTGTGACATTGACATCAATTGGTCTGGGAGATATTGTACCGAGAAGTGAAACCTATATGCTCATTACAATAGTCTACATTGCAATTGGTCTTGCTCTTACCACAATTGCCATTGAAATCGCCGCAGATGCATTGAAGAAGCTGCATTATTTTGGAAGAAAAATCGAAAATGTTGGAAATGTGGCTATATGGTTTGGAGGAAAGAAAATTACAATGAAAGCACTGGTCAAAAACCTCGGTGACCAGTTCAATCTTCCAACTACCGTAGTCAAGAATTTGAATTTGGATCATTTTGTGGATCAAGCGATTAAAGTGGAGGAAGGAGAGATTGAGACACTCAGACCGCCGCCTTATGAGCCACCGTCTGATCGATTTGAAGCTGAATTCGCTGATGAACCAGAATGTGAATGGATCCGTGATCCGACTCCAACTCCACCCCCATCACCTCAACCGGTTTATCGTCTTCCATCTCCAAAACCAGTTACACCGGAACCTCTACCAAGTCCAACAATAACTGATGTATCACTTGCGATTGCAACACCATCACCTGAAGAATCGGATGATGATCAAGAACTAATTCTCCCATCACCTGAACCGAGTCCAGTTCGAGAGCCAACTCCACCACCGCCTCCACGGGAGCCAACACCTCGTGAGCCAACTCCTGAGCCAGAGCCAGTTCGAGAGCCGACGCCTCCACCTCCGCCACCTGCCAAGCCCCGTCCACTGACTGCCGCTGAAATCGCGGCTCAAAAACGCAAAGCGTACAGCGAAGAAGCATGGCGTCGATACCAAGAATACCAGAAACAATGGAAGAAGTTCCGTCAAACTCAGAAAACTCCCGCACCATCTGGAGCCTCTACATCAGGGGCATCAACATCAAAACCGTCTGGAACATCACCGGAAAGTGGAGCCGGAGTATGTACTGGACCATCAACAAGGAGTCAATCAATAACATCAGTTGCATCTGGAAAGACATCAAGAAGTGCAACACCGGAAAGCAAGAAATCATCACATTTGAGTGGCTCATCGAGAAGAGAAAGCGGTGGAAAAGGATCCGGATCCGGATCCGGATCCGGAATGTCCGAGCTCATCAAGGAGAACATGCACATGAAGCTCTACATGGAGGGAACCGTCAACAACCACCACTTCAAGTGCACCTCCGAGGGAGAGGGAAAGCCATACGAGGGAACCCAAACCATGCGTATCAAGgtaagtttaaacatatatatactaactaaccctgattatttaaattttcagGCCGTCGAGGGAGGACCACTCCCATTCGCCTTCGACATCCTCGCCACCTCCTTCATGTACGGATCCAAGACCTTCATCAACCACACCCAAGGAATCCCAGACTTCTTCAAGCAATCCTTCCCAGAGGGATTCACCTGGGAGCGTGTCACCACCTACGAGGACGGAGGAGTCCTCACCGCCACCCAAGACACCTCCCTCCAAGACGGATGCCTCATCTACAACGTCAAGATCCGTGGAGTCAACTTCCCATCCAACGGACCAGTCATGCAAAAGAAGACCCTCGGATGGGAGGCCTCCACCGAGACCCTCTACCCAGCCGACGGAGGACTCGAGGGACGTGCCGACATGGCCCTCAAGCTCGTCGGAGGAGGACACCTCATCTGCAACCTCAAGgtaagtttaaacatgattttactaactaactaatctgatttaaattttcagACCACCTACCGTTCCAAGAAGCCAGCCAAGAACCTCAAGATGCCAGGAGTCTACTACGTCGACCGTCGTCTCGAGCGTATCAAGGAGGCCGACAAGGAGACCTACGTCGAGCAACACGAGGTCGCCGTCGCCCGTTACTGCGACCTCCCATCCAAGCTCGGACACCGTTAAaaatcatatgtttttctctctcacactctcttttttc

PHX2193: *flp-11(syb2193[flp-11b-SL2(gpd-2)-mKate2-linker-twk-18(e1913)])*X

>flp-11b- SL2(gpd-2)-mKate2(two introns)-linker(GSGSGSGSG)- twk-18(e1913)(one intron)

gtaagttgtcttaaaatttttcttccgctttttgcctttgcttcatgtgtcgtttattttgctttgcagttcgctttggccgatccggtcaactcgaccacatgcacgaccttttgtcgactcttcagAAGCTCAAGTTCGCCAACAACAAGTAATGACCGAGGACGACCGTCTTCTGCTCGAACAACTCCTG**CGA**CGAATTCATCATTAAgctgtctcatcctactttcacctagttaactgcttgtcttaaaatctatgcttctctttagtatctaaaattttcctagaagcttacaagtatataaatggtctcttctcaataaaggttgtatatttattcatcttattgaatctgccatttcctcgtttttgcgagtttatataccttccaattttctttctattgtattttcaacttctaattttaattcagggaaactgcttcaacgcatcaaaaATGTCCGAGCTCATCAAGGAGAACATGCACATGAAGCTCTACATGGAGGGAACCGTCAACAACCACCACTTCAAGTGCACCTCCGAGGGAGAGGGAAAGCCATACGAGGGAACCCAAACCATGCGTATCAAGgtaagtttaaacatatatatactaactaaccctgattatttaaattttcagGCCGTCGAGGGAGGACCACTCCCATTCGCCTTCGACATCCTCGCCACCTCCTTCATGTACGGATCCAAGACCTTCATCAACCACACCCAAGGAATCCCAGACTTCTTCAAGCAATCCTTCCCAGAGGGATTCACCTGGGAGCGTGTCACCACCTACGAGGACGGAGGAGTCCTCACCGCCACCCAAGACACCTCCCTCCAAGACGGATGCCTCATCTACAACGTCAAGATCCGTGGAGTCAACTTCCCATCCAACGGACCAGTCATGCAAAAGAAGACCCTCGGATGGGAGGCCTCCACCGAGACCCTCTACCCAGCCGACGGAGGACTCGAGGGACGTGCCGACATGGCCCTCAAGCTCGTCGGAGGAGGACACCTCATCTGCAACCTCAAGgtaagtttaaacatgattttactaactaactaatctgatttaaattttcagACCACCTACCGTTCCAAGAAGCCAGCCAAGAACCTCAAGATGCCAGGAGTCTACTACGTCGACCGTCGTCTCGAGCGTATCAAGGAGGCCGACAAGGAGACCTACGTCGAGCAACACGAGGTCGCCGTCGCCCGTTACTGCGACCTCCCATCCAAGCTCGGACACCGTGGATCCGGATCCGGATCCGGATCCGGAATGGCCATCGTCGCCCAAGGAGTCTCCACCATCCTCACCACCTTCCAAAAGACCTTCAAGGGACTCCTCCCACTCATCATCCTCGTCGCCTACACCCTCCTCGGAGCCTGGATCTTCTGGATGATCGAGGGAGAGAACGAGCGTGAGATGCTCATCGAGCAACAAAAGGAGCGTGACGAGCTCATCCGTCGTACCGTCTACAAGATCAACCAACTCCAAATCAAGCGTCAACGTCGTCTCATGACCGCCGAGGAGGAGTACAACCGTACCGCCAAGGTCCTCACCACCTTCCAAGAGACCCTCGGAATCGTCCCAGCCGACATGGACAAGGACATCCACTGGACCTTCCTCGGATCCATCTTCTACTGCATGACCGTCTACACCACCATCGGATACGGAAACATCGTCCCAGGAACCGGATGGGGACGTTTCGCCACCATCCTCTACGCCTTCATCGGAATCCCACTCACCGTCCTCTCCCTCTACTGCCTCGACTCCCTCTTCGCCAAGGGATGCAAGATGCTCTGGCGTTTCTTCCTCAAGTCCACCCGTGTCGTCTCCAAGGACCTCTCCAACAAGATCTCCGAGGCCGCCGACAACATCGAGGAGGGAACCACCGCCATCACCCCATCCGCCGAGAAGgtaagtttaaacagttcggtactaactaaccatacatatttaaattttcagACCGAGAACAACGACGACGACCTCCTCTCCTTCCCAATCTCCGGACTCCTCCTCATCACCGTCATCTGGGTCATCTTCTGCGCCGTCCTCTTCACCTTCCTCGAGGAGTGGGACTTCGGAACCTCCCTCTACTTCACCCTCATCTCCTTCACCACCATCGGATTCGGAGACATCCTCCCATCCGACTACGACTTCATGCCAATCGTCGGAGTCCTCCTCCTCATCGGACTCTCCCTCGTCTCCACCGTCATGACCCTCATCCAACAACAAATCGAGGCCCTCGCCTCCGGAATGAAGGACAACATCGACCAAGAGTACGCCCGTGCCCTCAACGAGGCCCGTGAGGACGGAGAGGTCGACGAGCACGTCGACCCAGAGGAGGACCCAGAGAACAACAAGAAGTCCTTCGACGCCGTCATCTCCCGTATGAACTGGTCCAAGCGTGGACTCTACTACCTCCTCCCAGACTCCCAAAAGAAGGAGCTCGCCAAGCAATCCGAGAAGAAGATGGGACGTAAGTCCATCAAGATCCAAACCGACAACGACCTCCTCGAGACCCTCATCCGTGAGGAGATCCTCAAGGCCGAGCTCAACAACGAGATGCACAAGTACACCGCCCCACGTTCCTCCCACCAACCAAAGCTCGTCTACTCCGACGTCCGTGAGAAGGAGGTCCCAATCGAGGTCGTCCGTGTCGAGCACTTCAACCACGGAAACGAGGACTACCTCGAGCACGACATCTAAaaatcatatgtttttctctctcacactctcttttttc

PHX2493: *lgc-38(syb2346[pflp-11::dpy-10 site::flp-11 3’UTR], syb2493[ReaChR-*

*linker-mKate2])*III

>ReaChR-linker-mKate2 in HB33(flp-11-5’utr::dpy-10 Crispr site::flp-11b-3’utr Chr III) pre lgc-38

tagcttttccttcctttccgaaatttaatgctattttcaagatgacttttttgcttgcgtttttctcagtttcctcacacacacacacacacaagtaggcgtggcctgtggaacgtttcagagcgcagaacacctgcatttgatctattcacttcttgcttttgaaaagcccaaagacaccctacacttcggtttcgttttggaaaccattgacatcatcctattttccataagaagtttccttgagaagaatccatttcgcaaatttttcattaaaacgttcaaaactcatcaaaccatttgtaaatagtaataaagtatgtcctgcggctatttgctttctcttcggaatctacaacgccccctcctaatacatcgtttcaggtataaaaagactgcgcctagccgctcgtctcactttttgcagttcatactgaata*aaaaa*ATGGTCTCCCGTCGTCCATGGCTCCTCGCCCTCGCCCTCGCCGTCGCCCTCGCCGCCGGATCCGCCGGAGCCTCCACCGGATCCGACGCCACCGTCCCAGTCGCCACCCAAGACGGACCAGACTACGTCTTCCACCGTGCCCACGAGCGTATGCTCTTCCAAACCTCCTACACCCTCGAGAACAACGGATCCGTCATCTGCATCCCAAACAACGGACAATGCTTCTGCCTCGCCTGGCTCAAGTCCAACGGAACCAACGCCGAGAAGCTCGCCGCCAACATCCTCCAATGGGTCGTCTTCGCCCTCTCCGTCGCCTGCCTCGGATGGTACGCCTACCAAGCCTGGCGTGCCACCTGCGGATGGGAGGAGGTCTACGTCGCCCTCATCGAGATGATGAAGTCCATCATCGAGGCCTTCCACGAGTTCGACTCCCCAGCCACCCTCTGGCTCTCCTCCGGAAACGGAGTCGTCTGGATGCGTTACGGAGAGTGGCTCCTCACCTGCCCAGTCATCCTCATCCACCTCTCCAACCTCACCGGACTCAAGgtaagtttaaacatatatatactaactaaccctgattatttaaattttcagGACGACTACTCCAAGCGTACCATGGGACTCCTCGTCTCCGACGTCGGATGCATCGTCTGGGGAGCCACCTCCGCCATGTGCACCGGATGGACCAAGATCCTCTTCTTCCTCATCTCCCTCTCCTACGGAATGTACACCTACTTCCACGCCGCCAAGGTCTACATCGAGGCCTTCCACACCGTCCCAAAGGGACTCTGCCGTCAACTCGTCCGTGCCATGGCCTGGCTCTTCTTCGTCTCCTGGGGAATGTTCCCAGTCCTCTTCCTCCTCGGACCAGAGGGATTCGGACACATCTCCCCATACGGATCCGCCATCGGACACTCCATCCTCGACCTCATCGCCAAGgtaagtttaaacagttcggtactaactaaccatacatatttaaattttcagAACATGTGGGGAGTCCTCGGAAACTACCTCCGTGTCAAGATCCACGAGCACATCCTCCTCTACGGAGACATCCGTAAGAAGCAAAAGATCACCATCGCCGGACAAGAGATGGAGGTCGAGACCCTCGTCGCCGAGGAGGAGGACAAGTACGAGTCCTCCGGAGGATCCGGAGGAGGATCCGGAGGAATGTCCGAGCTCATCAAGGAGAACATGCACATGAAGCTCTACATGGAGGGAACCGTCAACAACCACCACTTCAAGTGCACCTCCGAGGGAGAGGGAAAGCCATACGAGGGAACCCAAACCATGCGTATCAAGGCCGTCGAGGGAGGACCACTCCCATTCGCCTTCGACATCCTCGCCACCTCCTTCATGTACGGATCCAAGgtaagtttaaacatgattttactaactaactaatctgatttaaattttcagACCTTCATCAACCACACCCAAGGAATCCCAGACTTCTTCAAGCAATCCTTCCCAGAGGGATTCACCTGGGAGCGTGTCACCACCTACGAGGACGGAGGAGTCCTCACCGCCACCCAAGACACCTCCCTCCAAGACGGATGCCTCATCTACAACGTCAAGATCCGTGGAGTCAACTTCCCATCCAACGGACCAGTCATGCAAAAGAAGACCCTCGGATGGGAGGCCTCCACCGAGACCCTCTACCCAGCCGACGGAGGACTCGAGGGACGTGCCGACATGGCCCTCAAGCTCGTCGGAGGAGGACACCTCATCTGCAACCTCAAGACCACCTACCGTTCCAAGAAGCCAGCCAAGAACCTCAAGATGCCAGGAGTCTACTACGTCGACCGTCGTCTCGAGCGTATCAAGGAGGCCGACAAGGAGACCTACGTCGAGCAACACGAGGTCGCCGTCGCCCGTTACTGCGACCTCCCATCCAAGCTCGGACACCGTTAAaaatcatatgtttttctctctcacactctcttttttcatactctctcttgctgtctagaatttgattggtgtcgcttaacccccctttccctccgaaggaaagttatctccccagatctcttttggtgttttttatcagctaacaacacacattttctgatatttctatgctctgtctatgaacaataaaggcgttgttaattactcgcaaaatcactttgtttatttttttcacattttcagatagtgaacaaaagaaaattaaattctaaaatctgaatcggaaaattcaaattaaaaattaaatttattttttttatattacacctgttttttttcaaatattagatcaaaaactattcaacaagtggcatgtaaagcata**acg**aggtatatgggcttcagatcttcaactg

PHX3190: *lgc-38(syb2346[pflp-11::dpy-10 site::flp-11 3’UTR], syb2493[[unc- 58(e665)-linker-mKate2])*III

>unc-58(e665)-linker(GSGSGSGSG)-mKate2 in HB33(flp-11-5’utr::dpy-10 Crispr site::flp-11b-3’utr Chr III) pre lgc-38

cactttttgcagttcatactgaata*aaaaa*ATGGCTCCACTGACTGTGAAAAGCTCACCTCCAAAAAAGGCAAAAGGAATATCAAAATTTCGGAGAAAAAAGAAGCAGCCACCACCAGACTCAACCGTATTCGTCGCATGGGCACTCCGAAGTGTCCGAGAGTCTCTGATCCAAGTTGATCCATTGGCCGCTGCACTTGCACATCAGGCTCGAAAGACAAATAGTGTGCCAGCTGTCTCGAGAACTCCACTGCTTCTACAGTTCACTCCTTTCGGACCACCTCTCAGTGCGTATCATGTGACAGCTCGGTGGGAAGGTGCAAATATCAATTCACAATCAGCATTGCTCGATGCAGATGATGGAGCTACAGTTATCACAGATACCATCAAAGATGACCAAGATGATAAAGAACCAAAAAGCTGCCCGCAACAGACTGTCAAATACATCAAAATACTTACACCTCACGTGATCTTGGTGTCAGTGTTAATTGGATATTTATGCTTGGGAGCTTGGATACTCATGTTACTGGAAACAAGGACGGAACTTCTTGCCAGATCCAAAAAACTTGTCAGGTTAACAAATTTGATGTCAAACTTCACTGCCGAAAGTTGGAAGATGCTCAATAATGCTCAACACGGGGTTAGTAATATGGATGAAGGTGAATGGGCTGCAACATTTCGAGAATGGATGGTACGAGTATCAGAAACAGTGGACGATAGGAGACCTATACGACGTGAATTAAACCGGCCTGATGACTTATCAAATATGCATAATAAATGGACATTTCCAACTGCAATATTATATGTTCTCACTGTGTTAACTACTTGCGGTTATGGAGAAGTATCTGTCGACACAGACGTCGGAAAGGTTTTCTCAGTAGCATTCGCGCTTGTTGGTATACCACTTATGTTCATAACAGCTGCCGATATTGGTAAATTTTTATCTGAAACATTACTCCAGTTTGTGAGCTTTTGGAATCGAAGTGTCCGAAAAGTGAAGCAATGGATGAGTCGTATTCGTCACGGCAGGAGAAAGTCATTACAATCAACGGGTGGTCCCAACGATACTCTCGATATTCTTGGTGTCGACGGAACTGAAGAGAAACTTTGGTTCCCAATAGGTGCATATGTATCATGTATTTGCATATATTGCTCAATTGGGTCTGCCATGTTTATCACATGGGAAAGAACTTGGTCTTTCATTCATGCGTTTCATTTTGGTTTCAATTTGATTGTAACAGTCGGACTCGGAGATATCGTTGTGACTGATTACATATTTTTATCACTTATCGTTGCATTTGTGATAGTTGGTTTTTCCGTAGTGACCATGTGCGTGGATCTTGCGTCCACACATCTCAAGGCGTACTTCACCAGAATTCACTACTTTGGTCGAGCAAAACGATTCTTAGGAATGAGTGAGGAACTCAAAGAAATCGTTGCTTTACTGGGGGCGATGCGACGGAAAAAAGGCGGTAAAGTTACATGGAATGATGTGCGAGACTTCCTGGATAACGAACTCCGCGATCGACCTTTTGAACCTCATGAGCTTCTGATGAAGCTCAGATTTATTGACGAAACATCTTCTGGAATGTCTACAATCCGTCACAATTCCTTCCAGTCAGATTTTTTCCGAGAATCAGAGTACATCCGAAGAGTGGCTGCGCTGAGGCCAGAACAGCCAGCATATTTGGGATCCGGATCCGGATCCGGATCCGGAATGTCCGAGCTCATCAAGGAGAACATGCACATGAAGCTCTACATGGAGGGAACCGTCAACAACCACCACTTCAAGTGCACCTCCGAGGGAGAGGGAAAGCCATACGAGGGAACCCAAACCATGCGTATCAAGgtaagtttaaacatatatatactaactaaccctgattatttaaattttcagGCCGTCGAGGGAGGACCACTCCCATTCGCCTTCGACATCCTCGCCACCTCCTTCATGTACGGATCCAAGACCTTCATCAACCACACCCAAGGAATCCCAGACTTCTTCAAGCAATCCTTCCCAGAGGGATTCACCTGGGAGCGTGTCACCACCTACGAGGACGGAGGAGTCCTCACCGCCACCCAAGACACCTCCCTCCAAGACGGATGCCTCATCTACAACGTCAAGATCCGTGGAGTCAACTTCCCATCCAACGGACCAGTCATGCAAAAGAAGACCCTCGGATGGGAGGCCTCCACCGAGACCCTCTACCCAGCCGACGGAGGACTCGAGGGACGTGCCGACATGGCCCTCAAGCTCGTCGGAGGAGGACACCTCATCTGCAACCTCAAGgtaagtttaaacatgattttactaactaactaatctgatttaaattttcagACCACCTACCGTTCCAAGAAGCCAGCCAAGAACCTCAAGATGCCAGGAGTCTACTACGTCGACCGTCGTCTCGAGCGTATCAAGGAGGCCGACAAGGAGACCTACGTCGAGCAACACGAGGTCGCCGTCGCCCGTTACTGCGACCTCCCATCCAAGCTCGGACACCGTTAAaaatcatatgtttttctctctcacactctc

PHX4110: *lgc-38(syb2346[flp-11p::dpy-10 site::flp-11 3’UTR], syb4110[unc-58gf-CAI-1.0-linker(GSGSGSGSG)-mKate2]) III*

>unc-58(e665CAI)-linker(GSGSGSGSG)-mKate2 in HB33(flp-11-5’utr::dpy-10 Crispr site::flp-11b-3’utr Chr III) pre lgc-38

cactttttgcagttcatactgaata*aaaaa*ATGGCCCCACTCACCGTCAAGTCCTCCCCACCAAAGAAGGCCAAGGGAATCTCCAAGTTCCGTCGTAAGAAGAAGCAACCACCACCAGACTCCACCGTCTTCGTCGCCTGGGCCCTCCGTTCCGTCCGTGAGTCCCTCATCCAAGTCGACCCACTCGCCGCCGCCCTCGCCCACCAAGCCCGTAAGACCAACTCCGTCCCAGCCGTCTCCCGTACCCCACTCCTCCTCCAATTCACCCCATTCGGACCACCACTCTCCGCCTACCACGTCACCGCCCGTTGGGAGGGAGCCAACATCAACTCCCAATCCGCCCTCCTCGACGCCGACGACGGAGCCACCGTCATCACCGACACCATCAAGGACGACCAAGACGACAAGGAGCCAAAGTCCTGCCCACAACAAACCGTCAAGTACATCAAGATCCTCACCCCACACGTCATCCTCGTCTCCGTCCTCATCGGATACCTCTGCCTCGGAGCCTGGATCCTCATGCTCCTCGAGACCCGTACCGAGCTCCTCGCCCGTTCCAAGAAGCTCGTCCGTCTCACCAACCTCATGTCCAACTTCACCGCCGAGTCCTGGAAGATGCTCAACAACGCCCAACACGGAGTCTCCAACATGGACGAGGGAGAGTGGGCCGCCACCTTCCGTGAGTGGATGGTCCGTGTCTCCGAGACCGTCGACGACCGTCGTCCAATCCGTCGTGAGCTCAACCGTCCAGACGACCTCTCCAACATGCACAACAAGTGGACCTTCCCAACCGCCATCCTCTACGTCCTCACCGTCCTCACCACCTGCGGATACGGAGAGGTCTCCGTCGACACCGACGTCGGAAAGGTCTTCTCCGTCGCCTTCGCCCTCGTCGGAATCCCACTCATGTTCATCACCGCCGCCGACATCGGAAAGTTCCTCTCCGAGACCCTCCTCCAATTCGTCTCCTTCTGGAACCGTTCCGTCCGTAAGGTCAAGCAATGGATGTCCCGTATCCGTCACGGACGTCGTAAGTCCCTCCAATCCACCGGAGGACCAAACGACACCCTCGACATCCTCGGAGTCGACGGAACCGAGGAGAAGCTCTGGTTCCCAATCGGAGCCTACGTCTCCTGCATCTGCATCTACTGCTCCATCGGATCCGCCATGTTCATCACCTGGGAGCGTACCTGGTCCTTCATCCACGCCTTCCACTTCGGATTCAACCTCATCGTCACCGTCGGACTCGGAGACATCGTCGTCACCGACTACATCTTCCTCTCCCTCATCGTCGCCTTCGTCATCGTCGGATTCTCCGTCGTCACCATGTGCGTCGACCTCGCCTCCACCCACCTCAAGGCCTACTTCACCCGTATCCACTACTTCGGACGTGCCAAGCGTTTCCTCGGAATGTCCGAGGAGCTCAAGGAGATCGTCGCCCTCCTCGGAGCCATGCGTCGTAAGAAGGGAGGAAAGGTCACCTGGAACGACGTCCGTGACTTCCTCGACAACGAGCTCCGTGACCGTCCATTCGAGCCACACGAGCTCCTCATGAAGCTCCGTTTCATCGACGAGACCTCCTCCGGAATGTCCACCATCCGTCACAACTCCTTCCAATCCGACTTCTTCCGTGAGTCCGAGTACATCCGTCGTGTCGCCGCCCTCCGTCCAGAGCAACCAGCCTACCTCGGATCCGGATCCGGATCCGGATCCGGAATGTCCGAGCTCATCAAGGAGAACATGCACATGAAGCTCTACATGGAGGGAACCGTCAACAACCACCACTTCAAGTGCACCTCCGAGGGAGAGGGAAAGCCATACGAGGGAACCCAAACCATGCGTATCAAGgtaagtttaaacatatatatactaactaaccctgattatttaaattttcagGCCGTCGAGGGAGGACCACTCCCATTCGCCTTCGACATCCTCGCCACCTCCTTCATGTACGGATCCAAGACCTTCATCAACCACACCCAAGGAATCCCAGACTTCTTCAAGCAATCCTTCCCAGAGGGATTCACCTGGGAGCGTGTCACCACCTACGAGGACGGAGGAGTCCTCACCGCCACCCAAGACACCTCCCTCCAAGACGGATGCCTCATCTACAACGTCAAGATCCGTGGAGTCAACTTCCCATCCAACGGACCAGTCATGCAAAAGAAGACCCTCGGATGGGAGGCCTCCACCGAGACCCTCTACCCAGCCGACGGAGGACTCGAGGGACGTGCCGACATGGCCCTCAAGCTCGTCGGAGGAGGACACCTCATCTGCAACCTCAAGgtaagtttaaacatgattttactaactaactaatctgatttaaattttcagACCACCTACCGTTCCAAGAAGCCAGCCAAGAACCTCAAGATGCCAGGAGTCTACTACGTCGACCGTCGTCTCGAGCGTATCAAGGAGGCCGACAAGGAGACCTACGTCGAGCAACACGAGGTCGCCGTCGCCCGTTACTGCGACCTCCCATCCAAGCTCGGACACCGTTAAaaatcatatgtttttctc

PHX4416 *aptf-1(gk794)II; flp-11(syb1445 syb4416) X*

>flp-11promoter-unc-58(e665)-linker(GSGSGSGSG)-mKate2(two introns)
tctcttcggaatctacaacgccccctcctaatacatcgtttcaggtataaaaagactgcgcctagccgctcgtctcactttttgcagttcatactgaataATGGCTCCACTGACTGTGAAAAGCTCACCTCCAAAAAAGGCAAAAGGAATATCAAAATTTCGGAGAAAAAAGAAGCAGCCACCACCAGACTCAACCGTATTCGTCGCATGGGCACTCCGAAGTGTCCGAGAGTCTCTGATCCAAGTTGATCCATTGGCCGCTGCACTTGCACATCAGGCTCGAAAGACAAATAGTGTGCCAGCTGTCTCGAGAACTCCACTGCTTCTACAGTTCACTCCTTTCGGACCACCTCTCAGTGCGTATCATGTGACAGCTCGGTGGGAAGGTGCAAATATCAATTCACAATCAGCATTGCTCGATGCAGATGATGGAGCTACAGTTATCACAGATACCATCAAAGATGACCAAGATGATAAAGAACCAAAAAGCTGCCCGCAACAGACTGTCAAATACATCAAAATACTTACACCTCACGTGATCTTGGTGTCAGTGTTAATTGGATATTTATGCTTGGGAGCTTGGATACTCATGTTACTGGAAACAAGGACGGAACTTCTTGCCAGATCCAAAAAACTTGTCAGGTTAACAAATTTGATGTCAAACTTCACTGCCGAAAGTTGGAAGATGCTCAATAATGCTCAACACGGGGTTAGTAATATGGATGAAGGTGAATGGGCTGCAACATTTCGAGAATGGATGGTACGAGTATCAGAAACAGTGGACGATAGGAGACCTATACGACGTGAATTAAACCGGCCTGATGACTTATCAAATATGCATAATAAATGGACATTTCCAACTGCAATATTATATGTTCTCACTGTGTTAACTACTTGCGGTTATGGAGAAGTATCTGTCGACACAGACGTCGGAAAGGTTTTCTCAGTAGCATTCGCGCTTGTTGGTATACCACTTATGTTCATAACAGCTGCCGATATTGGTAAATTTTTATCTGAAACATTACTCCAGTTTGTGAGCTTTTGGAATCGAAGTGTCCGAAAAGTGAAGCAATGGATGAGTCGTATTCGTCACGGCAGGAGAAAGTCATTACAATCAACGGGTGGTCCCAACGATACTCTCGATATTCTTGGTGTCGACGGAACTGAAGAGAAACTTTGGTTCCCAATAGGTGCATATGTATCATGTATTTGCATATATTGCTCAATTGGGTCTGCCATGTTTATCACATGGGAAAGAACTTGGTCTTTCATTCATGCGTTTCATTTTGGTTTCAATTTGATTGTAACAGTCGGACTCGGAGATATCGTTGTGACTGATTACATATTTTTATCACTTATCGTTGCATTTGTGATAGTTGGTTTTTCCGTAGTGACCATGTGCGTGGATCTTGCGTCCACACATCTCAAGGCGTACTTCACCAGAATTCACTACTTTGGTCGAGCAAAACGATTCTTAGGAATGAGTGAGGAACTCAAAGAAATCGTTGCTTTACTGGGGGCGATGCGACGGAAAAAAGGCGGTAAAGTTACATGGAATGATGTGCGAGACTTCCTGGATAACGAACTCCGCGATCGACCTTTTGAACCTCATGAGCTTCTGATGAAGCTCAGATTTATTGACGAAACATCTTCTGGAATGTCTACAATCCGTCACAATTCCTTCCAGTCAGATTTTTTCCGAGAATCAGAGTACATCCGAAGAGTGGCTGCGCTGAGGCCAGAACAGCCAGCATATTTGGGATCCGGATCCGGATCCGGATCCGGAATGTCCGAGCTCATCAAGGAGAACATGCACATGAAGCTCTACATGGAGGGAACCGTCAACAACCACCACTTCAAGTGCACCTCCGAGGGAGAGGGAAAGCCATACGAGGGAACCCAAACCATGCGTATCAAGgtaagtttaaacatatatatactaactaaccctgattatttaaattttcagGCCGTCGAGGGAGGACCACTCCCATTCGCCTTCGACATCCTCGCCACCTCCTTCATGTACGGATCCAAGACCTTCATCAACCACACCCAAGGAATCCCAGACTTCTTCAAGCAATCCTTCCCAGAGGGATTCACCTGGGAGCGTGTCACCACCTACGAGGACGGAGGAGTCCTCACCGCCACCCAAGACACCTCCCTCCAAGACGGATGCCTCATCTACAACGTCAAGATCCGTGGAGTCAACTTCCCATCCAACGGACCAGTCATGCAAAAGAAGACCCTCGGATGGGAGGCCTCCACCGAGACCCTCTACCCAGCCGACGGAGGACTCGAGGGACGTGCCGACATGGCCCTCAAGCTCGTCGGAGGAGGACACCTCATCTGCAACCTCAAGgtaagtttaaacatgattttactaactaactaatctgatttaaattttcagACCACCTACCGTTCCAAGAAGCCAGCCAAGAACCTCAAGATGCCAGGAGTCTACTACGTCGACCGTCGTCTCGAGCGTATCAAGGAGGCCGACAAGGAGACCTACGTCGAGCAACACGAGGTCGCCGTCGCCCGTTACTGCGACCTCCCATCCAAGCTCGGACACCGTTAAaaatcatatgtttttctctctcacactctcttttttcatactctctcttgctgtctagaatttgattggtgtcgcttaacccccctttccctccgaagga
