# Supplementary figures and images for: A sleep-active neuron can promote survival while sleep behavior is disturbed
Source: PLoS Genet. 2023 Mar 14;19(3):e1010665. doi: 10.1371/journal.pgen.1010665 (PMC10038310; doi:10.1371/journal.pgen.1010665)

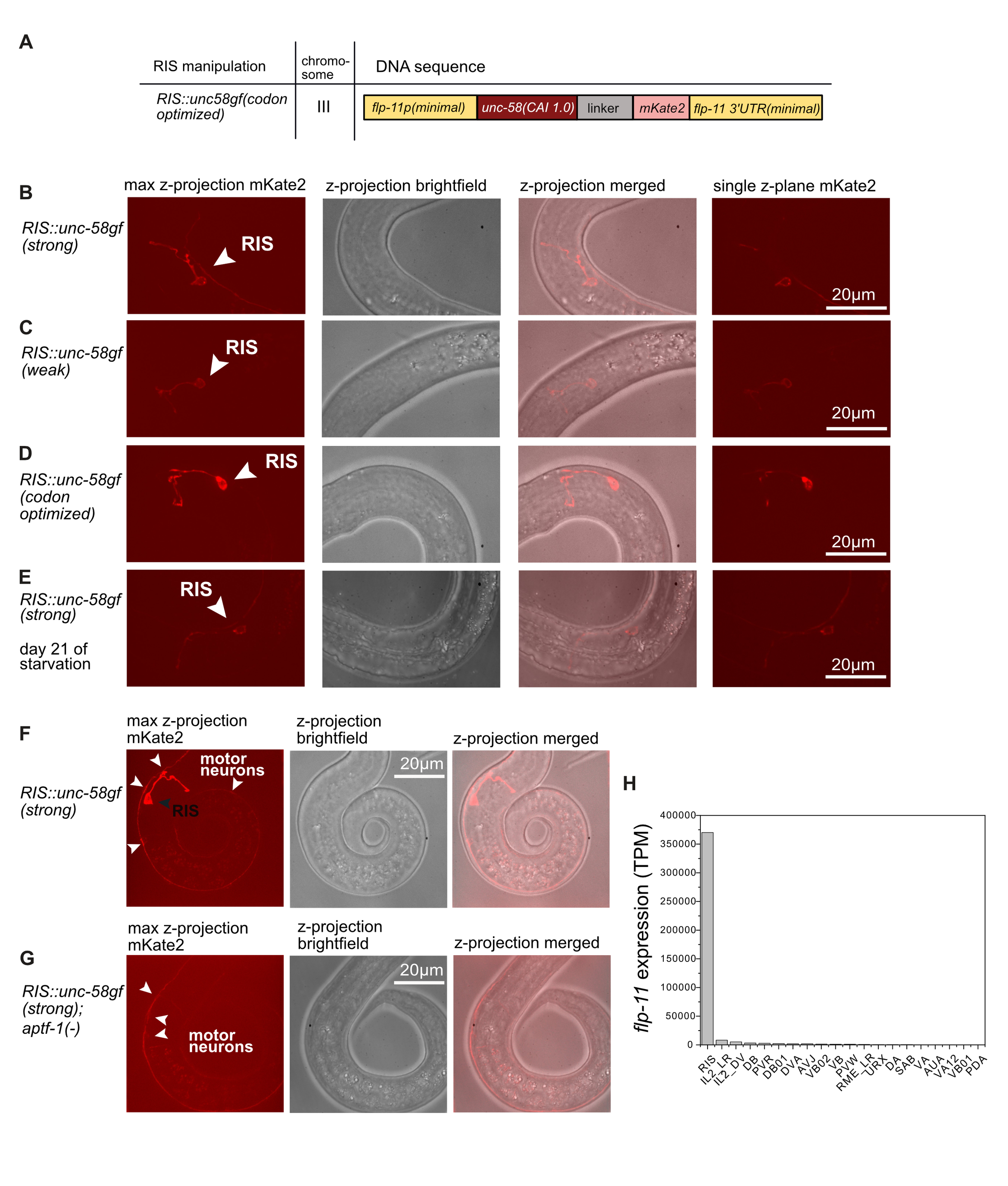

Supplement: S1 Fig — A) Scheme of the genetic design of the RIS::unc-58gf(weak) transgene. B-D) Localization and expression of the different RIS depolarization strains. E) On day 21 of starvation RIS appears to be intact in the strong depolarization strain (RIS::unc-58gf(strong)). F) Imaging conditions that oversaturate the mKate2 signal in RIS in the RIS::unc-58gf(strong) strain reveal a weak expression of the tool in neurons in the ventral cord. G) The weak expression in the ventral cord neurons but not in RIS is preserved in an aptf-1 deletion. H) Transcriptomic data from the Cengen project suggest that the weakly expressing neurons could be IL2 and motor neurons [31]. (TIF) [file pgen.1010665.s002.tif]

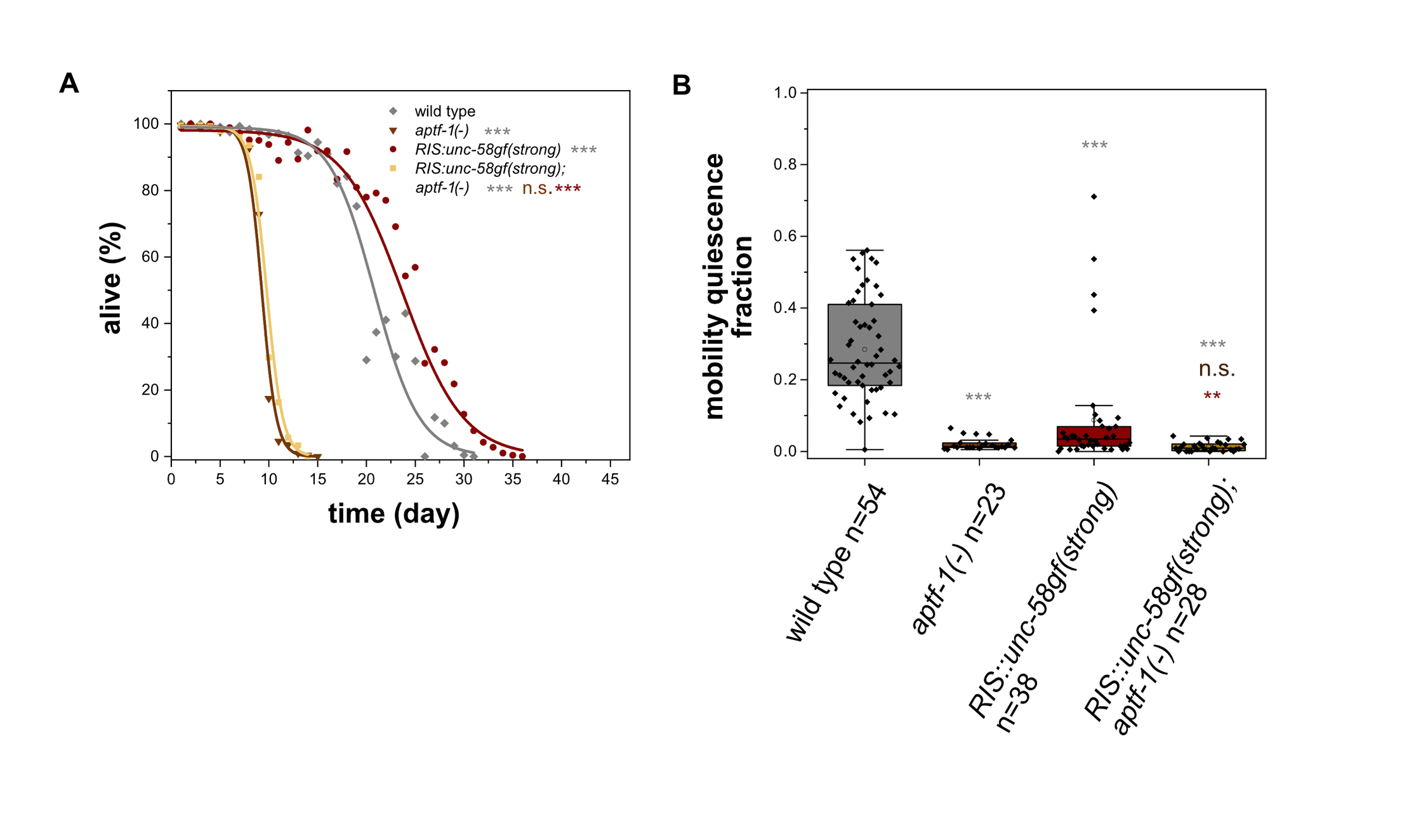

Supplement: S2 Fig — A) The survival benefit of RIS::unc-58gf(strong) strongly decreased in the RIS::unc-58gf(strong), aptf-1(-). Fisher’s Exact Test was conducted on day 11) when aptf-1(-) was the shortest-lived condition and on day 23) when wild type was the shortest-lived condition. The plot includes data from two replicates ***p<0.001. B) RIS::unc-58gf(strong) abolishes quiescence behavior in most individuals similar to but not as strongly as aptf-1(-). A few RIS::unc-58gf(strong) animals showed a high fraction of quiescence. To test whether the rare occurrence of increased quiescence phenotype stemmed from unc-58gf expression in RIS, we measured quiescence in an aptf-1(-) background. This completely abolished quiescence in all animals. (TIF) [file pgen.1010665.s003.tif]

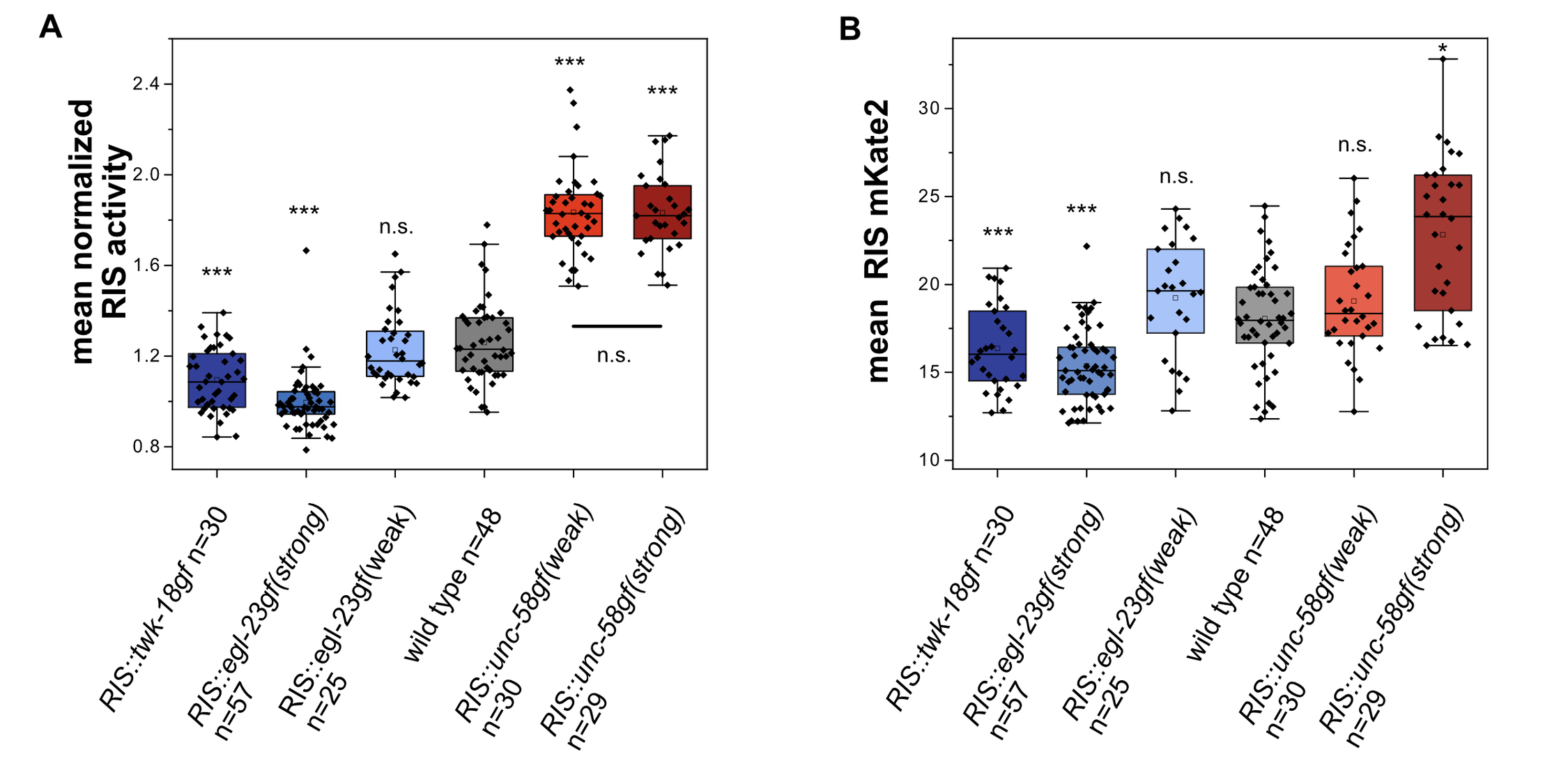

Supplement: S3 Fig — A) Mean RIS activity of individual worms of the different RIS activity strains. n.s. p>0.05, **p<0.001, ***p<0.001, Welch test with FDR correction for multiple testing. B) Mean RIS mKate2 intensities of individual worms as a transcriptional reporter. n.s. p>0.05, *p<0.05, ***p<0.001, Welch test with FDR correction for multiple testing. (TIF) [file pgen.1010665.s004.tif]

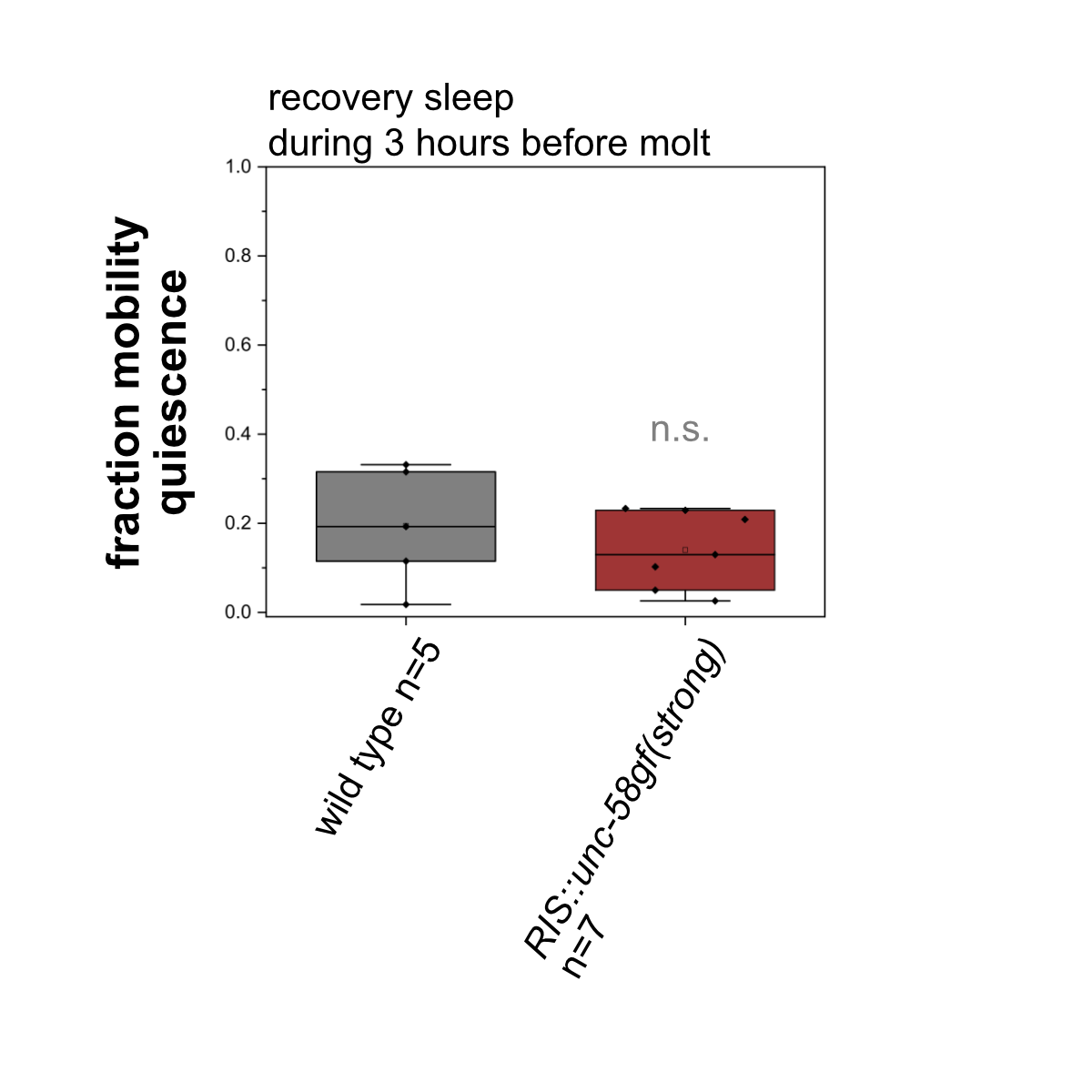

Supplement: S4 Fig — There was no detectable rebound sleep during L1 lethargus after worms were fed after 12 days of starvation. n.s. p>0.05, Welch test. (TIF) [file pgen.1010665.s005.tif]

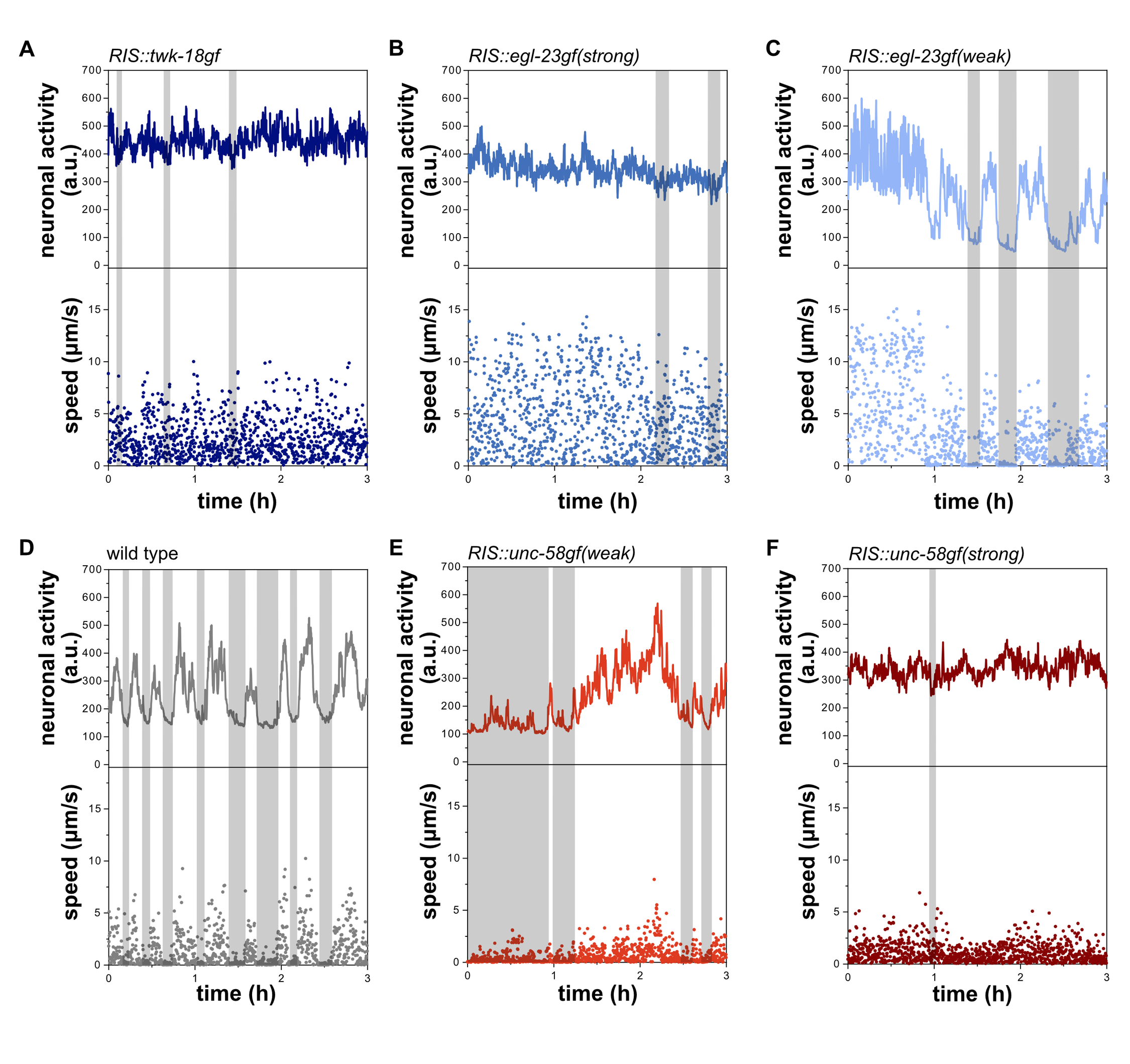

Supplement: S5 Fig — A-F) Sample traces of different RIS activity strains showing neuronal activity and speed after 48h starvation in L1 arrest. Neuronal inactivity bouts, which are depicted in a grey shade, strongly correlate with mobility quiescence. (TIF) [file pgen.1010665.s006.tif]

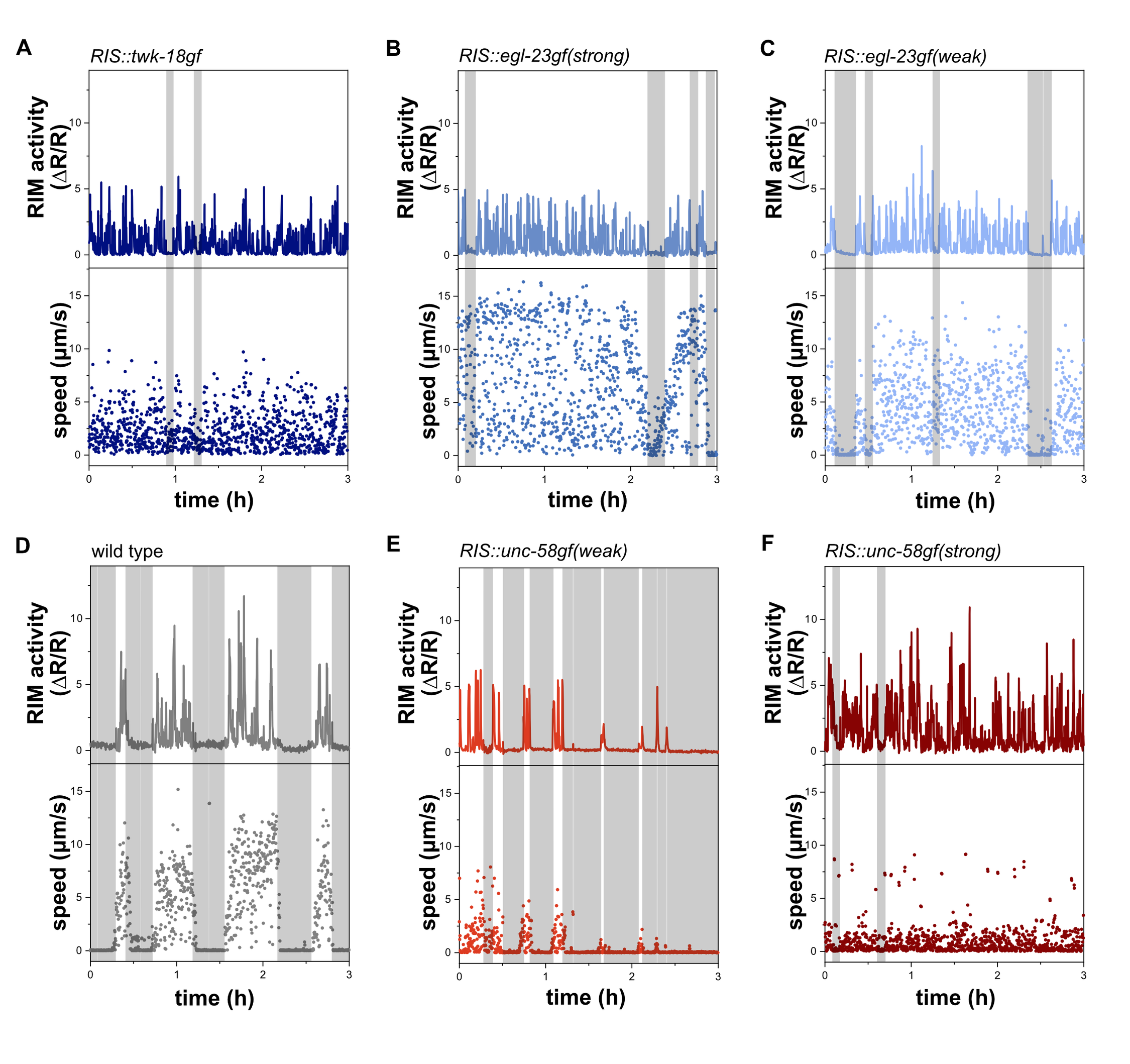

Supplement: S6 Fig — A-F) Sample traces of strains with different RIS activity are showing RIM activity and speed after 48h starvation in first larval stage arrest. Times of RIM inactivity (grey shaded area) and immobility strongly correlate. (TIF) [file pgen.1010665.s007.tif]

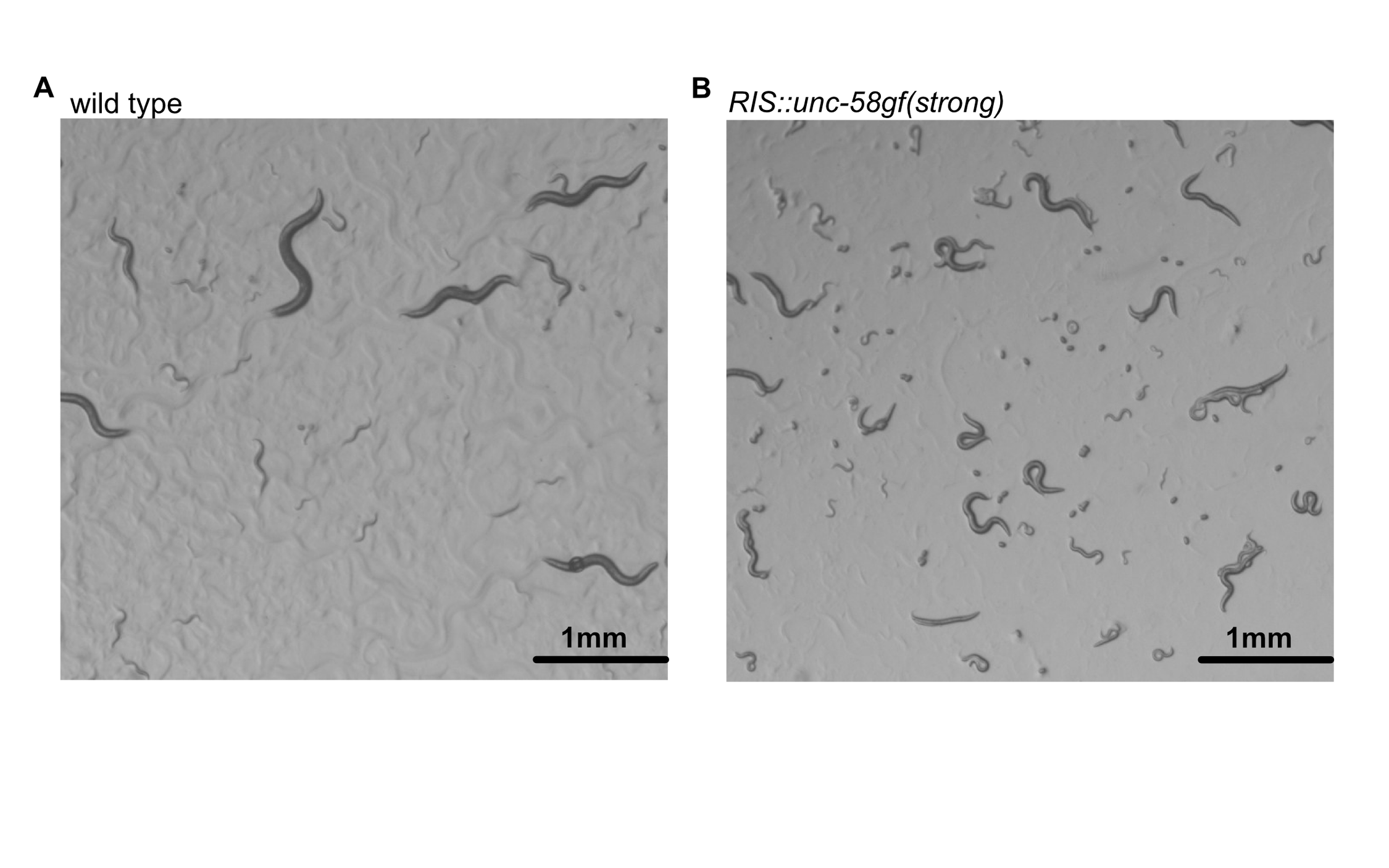

Supplement: S7 Fig — A) Image of wild-type worms on an NGM plate. B) Image of RIS::unc-58gf(strong) worms on an NGM plate. (TIF) [file pgen.1010665.s008.tif]

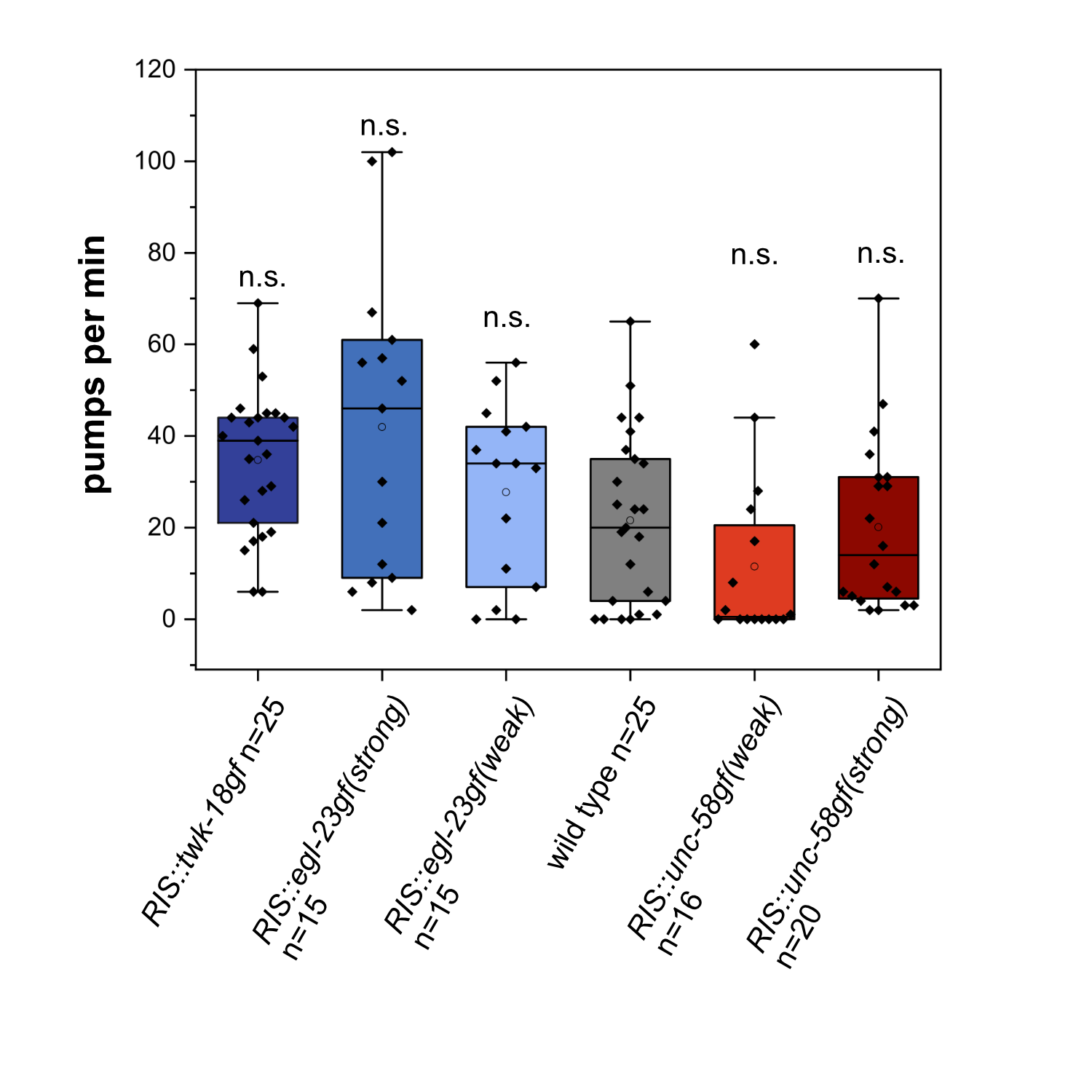

Supplement: S8 Fig — The pumping rate of RIS::unc-58gf(strong) in arrested L1 worms is comparable to the wild type. (TIF) [file pgen.1010665.s009.tif]

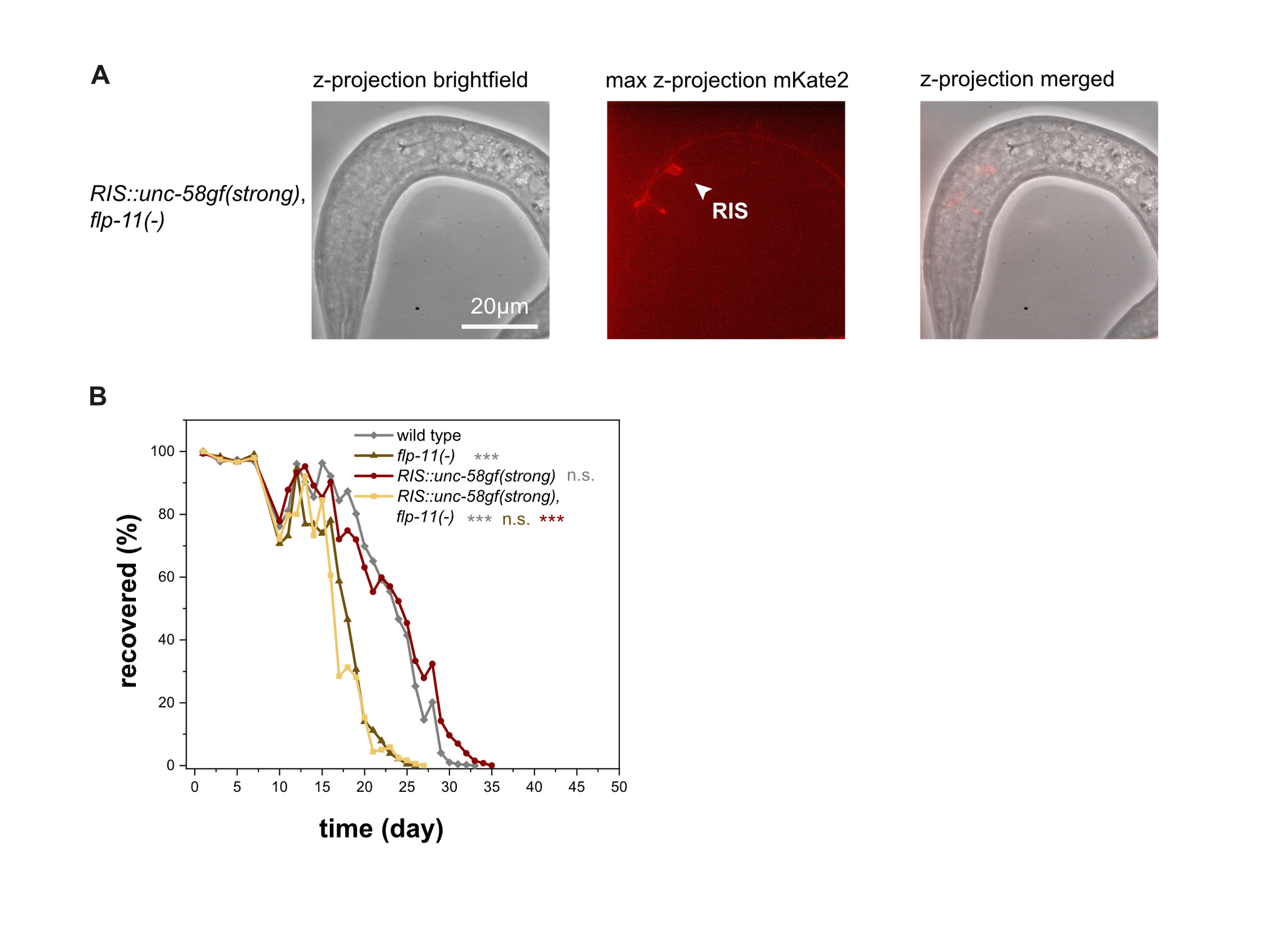

Supplement: S9 Fig — A) The UNC-58gf channel is still expressed at the plasma membrane of RIS in RIS::unc-58gf(strong), flp-11(-). B) flp-11(-) causes strongly reduced recovery rates (Fisher’s Exact Test was conducted on day 18) for comparisons when flp-11(-) was the shortest lived condition, day 20) when RIS::unc-58gf(strong), flp-11(-) was the shortest-lived condition or day 24) when wild type was the shortest-lived condition. The p-values were FDR corrected with Benjamini-Hochberg procedure with a 5% false discovery rate. The plot includes data from three replicates. ***p<0.001. (TIF) [file pgen.1010665.s010.tif]

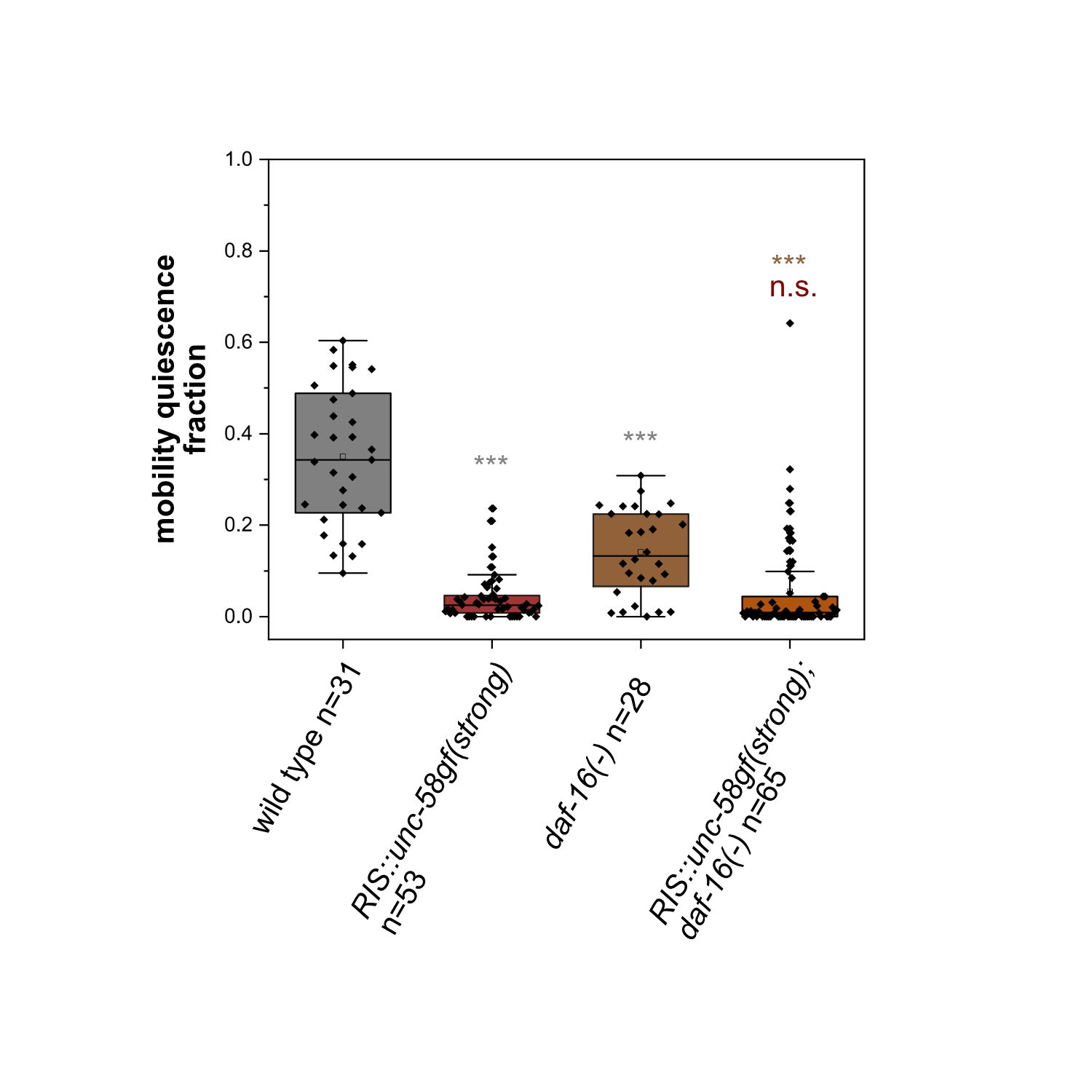

Supplement: S10 Fig — Loss of DAF-16 does not change mobility quiescence of RIS::unc-58gf(strong). (TIF) [file pgen.1010665.s011.tif]
