# Supplementary material for: A sleep-active neuron can promote survival while sleep behavior is disturbed
Source: PLoS Genet. 2023 Mar 14;19(3):e1010665. doi: 10.1371/journal.pgen.1010665 (PMC10038310; doi:10.1371/journal.pgen.1010665)
Supplement: S1 Table — (DOCX) [file pgen.1010665.s012.docx]

$$y= \frac{a}{1+e^{-k*(x-x_{c})}}$$

| Figure | strain average | a | k | x_c_ | R^2^ |
| --- | --- | --- | --- | --- | --- |
| 2B | RIS::twk-18gf | 99.35847 ± 0.66637 | -0.97206 ± 0.12292 | 10.82146 ± 0.33634 | 0.99956 |
| 2B | RIS::egl-23gf(strong) | 100.04807 ± 0.44986 | -0.48094 ± 0.02632 | 17.15292 ± 0.2396 | 0.99956 |
| 2B | RIS::egl-23gf(weak) | 97.32902 ± 0.63724 | -0.41377 ± 0.03302 | 21.60212 ± 0.40667 | 0.99842 |
| 2B | wild type | 98.76819 ± 0.81028 | -0.36143 ± 0.0284 | 21.87268 ± 0.44843 | 0.99803 |
| 2B | RIS::unc-58gf(weak) | 100.02342 ± 1.01326 | -0.24451 ± 0.01495 | 22.19817 ± 0.40979 | 0.99807 |
| 2B | RIS::unc-58gf(strong) | 98.12395 ± 0.74149 | -0.30653 ± 0.02368 | 25.51191 ± 0.46922 | 0.99738 |
| 12B | wild type | 98.96086± 0.83837 | -0.36524± 0.03139 | 22.58115± 0.48943 | 0.99735 |
| 12B | *flp-11(-)* | 98.56064± 0.71073 | -0.48376± 0.04287 | 17.90444± 0.39309 | 0.99859 |
| 12B | RIS:unc-58gf(strong) | 97.36395± 0.51755 | -0.34025± 0.02311 | 26.12587± 0.37504 | 0.99827 |
| 12B | RIS:unc-58gf(strong), *flp-11(-)* | 98.50514± 0.81164 | -0.38458± 0.03107 | 19.2531± 0.40608 | 0.99817 |
| S2A | RIS::unc-58gf(strong), *aptf-1(-)* | 99.28789 ± 0.30363 | -1.2192 ± 0.11022 | 9.78636± 0.20379 | 0.99985 |
| S2A | *aptf-1(-)* | 99.46017± 0.3177 | -0.32131± 0.0232 | 23.73166± 0.44811 | 0.99989 |
| S2A | RIS::unc-58gf(strong) | 98.15331± 0.66903 | -0.32131± 0.0232 | 23.73166± 0.44811 | 0.99774 |
| S2A | wild type | 99.01336± 0.50766 | -0.43604± 0.03443 | 20.83339± 0.37968 | 0.99867 |

3 parameter logistic fit for lifespans.
