# Supplementary material for: A sleep-active neuron can promote survival while sleep behavior is disturbed
Source: PLoS Genet. 2023 Mar 14;19(3):e1010665. doi: 10.1371/journal.pgen.1010665 (PMC10038310; doi:10.1371/journal.pgen.1010665)
Supplement: S2 Table — (DOCX) [file pgen.1010665.s013.docx]

CF1038 *daf-16(mu86) I*. CGC

HBR4 *goeIs3(pmyo-3p::SL1-GCamP3.35-SL2::unc54-3'utr, unc-119(+)).* [1]

HBR507 *flp-11(tm2706)* X. [2]

HBR560 *goeIs120 (tdc-1p::SL1-GCaMP3.35-SL2::mKate2-unc-54-3’utr, unc119(+)).* [3]

HBR923 *goeIs207(mec-4p::SL1-GCaMP6s::mkate2-unc-54-3'utr, unc-119(+)).*

HBR1361 *goeIs304(flp-11p::SL1-GCaMP3.35-SL2::mKate2-unc-54-3'UTR, unc-119(+)).* [4]

HBR2340 *flp-11(syb1445[flp-11-SL2-unc-58(L428F)-linker-mKate2])* X. Generated for this study by outcrossing PHX1445 with N2.

HBR2370 *flp-11(syb2193[flp-11-SL2(gpd-2)-mKate2-linker-twk-18(e1913)])* X*; goeIs304(flp-11p::SL1-GCaMP3.35-SL2::mKate2-unc-54-3'UTR, unc-119(+)).* Generated for this study by crossing HBR1361 with PHX2193.

HBR2371 *flp-11(syb1445[flp-11-SL2-unc-58(L428F)-linker-mKate2])* X*; goeIs304(flp-11p::SL1-GCaMP3.35-SL2::mKate2-unc-54-3'UTR.* Generated for this study by crossing HBR1361 with HBR2340.

HBR2421 *flp-11(syb1445[flp-11-SL2-unc-58(L428F)-linker-mKate2])* X*; otIs672(rab-3p::NLS::GCaMP6s, arrd-4p:NLS:::GCaMP6s).*

Generated for this study by crossing OH15265 with HBR2340.

HBR2446 *flp-11(syb1445[flp-11-SL2-unc-58(L428F)-linker-mKate2]) X, goeIs120(tdc-1p::SL1-GCaMP3.35-SL2::mKate2-unc-54-3'utr,unc119(+)).* Generated for this study by crossing PHX1445 and HBR560.

HBR2470 *flp-11(syb2193[flp-11b-SL2(gpd-2)-mKate2-linker-twk-18(e1913)]) X, otIs672(rab-3p::NLS::GCaMP6s, arrd-4p:NLS:::GCaMP6s).* Generated for this study by crossing PHX2193 and OH15265.

HBR2508 *flp-11(syb2193[flp-11b-SL2(gpd-2)-mKate2-linker-twk-18(e1913)]) X; goeIs120(tdc-1p::SL1-GCaMP3.35-SL2::mKate2-unc-54-3'utr, unc119(+)).* Generated for this study by crossing PHX2193 and HBR560.

HBR2522 *lgc-38(syb2346[flp-11p::dpy-10 site::flp-11 3’UTR], syb3190[unc-58(e665)-linker(GSGSGSGSG)-mKate2 ]) III; goeIs304(flp-11p::SL1-GCaMP3.35-SL2::mKate2-unc-54-3'UTR, unc-119(+)).* Generated for this study by crossing PHX3190 and HBR1361.

HBR2523 *lgc-38(syb2346[pflp-11::dpy-10 site::flp-11 3’UTR], syb2493[ReaChR-linker-mKate2]) III; goeIs304(pflp-11::SL1-GCaMP3.35-SL2::mKate2-unc-54-3'UTR, unc-119(+)).* Generated for this study by crossing HBR1361 and PHX2493.

HBR2540 *flp-11(syb1433[flp-11-SL2-egl-23cDNA(A383V)-linker-mKate2-N2]) X; otIs672(rab-3p::NLS::GCaMP6s, arrd-4p:NLS:::GCaMP6s).* Generated for this study by crossing PHX1433 and OH15265.

HBR2541 *flp-11(syb1464)[flp-11-SL2-egl-23cDNA(L229N)-linker-mKate2-N2]) X; otIs672(rab-3p::NLS::GCaMP6s, arrd-4p:NLS:::GCaMP6s).* Generated for this study by crossing PHX1464 and OH15265.

HBR2542 *flp-11(syb1433[flp-11-SL2-egl-23cDNA(A383V)-linker-mKate2-N2]) X; goeIs304(flp-11p::SL1-GCaMP3.35-SL2::mKate2-unc-54-3'UTR, unc-119(+)).* Generated for this study by crossing PHX1433 and HBR1361.

HBR2543 *flp-11(syb1464)[flp-11-SL2-egl-23cDNA(L229N)-linker-mKate2-N2]) X; goeIs304(flp-11p::SL1-GCaMP3.35-SL2::mKate2-unc-54-3'UTR, unc-119(+)).* Generated for this study by crossing PHX1464 and HBR1361.

HBR2544 *lgc-38(syb2346[flp-11p::dpy-10 site::flp-11 3’UTR] III, syb3190[unc-58(e665)-linker(GSGSGSGSG)-mKate2]); goeIs120(tdc-1p::SL1-GCaMP3.35-SL2::mKate2-unc-54-3'utr,unc119(+)).* Generated for this study by crossing PHX3190 and HBR560.

HBR2545 *daf-16(mu86) I; flp-11(syb1445[flp-11-SL2-unc-58(L428F)-linker-mKate2])* X. [5]

HBR2584 *lgc-38(syb2346[flp-11p::dpy-10 site::flp-11 3’UTR], syb2493[ReaChR-linker-mKate2])* III *; flp-11(syb1445[flp-11-SL2-unc-58(L428F)-linker-mKate2])* X*; goeIs304(flp-11p::SL1-GCaMP3.35-SL2::mKate2-unc-54-3'UTR.* Generated for this study by crossing HBR2371 and PHX2493.

HBR2587 *flp-11(syb1433[flp-11-SL2-egl-23cDNA(A383V)-linker-mKate2-N2]) X; goeIs120(tdc-1p::SL1-GCaMP3.35-SL2::mKate2-unc-54-3'utr, unc119(+)).* Generated for this study by crossing HBR560 and PHX1433.

HBR2628 *flp-11(syb1464[flp-11-SL2-egl-23cDNA(L229N)-linker-mKate2-N2]) X; goeIs120(tdc-1p::SL1-GCaMP3.35-SL2::mKate2-unc-54-3'utr, unc119(+))*. Generated for this study by crossing HBR560 and PHX1464.

HBR2629 *lgc-38(syb2346[flp-11p::dpy-10 site::flp-11 3’UTR], syb3190[unc-58(e665)-linker(GSGSGSGSG)-mKate2]) III, otIs672(rab-3p::NLS::GCaMP6s, arrd-4p:NLS:::GCaMP6s).* Generated for this study by crossing PHX3190 and OH15265.

HBR2652 *goeIs207(mec-4p::SL1-GCaMP6s::mkate2-unc-54-3'utr, unc-119(+)); flp-11(syb2193[flp-11-SL2-mKate2-linker-twk-18(e1913)])* X*.* Generated for this study by crossing PHX2193 with HBR923.

HBR2656 *goeIs207(mec-4p::SL1-GCaMP6s::mkate2-unc-54-3'utr, unc-119(+)); flp-11(syb1445[flp-11-SL2-unc-58(L428F)-linker-mKate2])* X. Generated for this study by crossing HBR2340 and HBR923.

HBR2658 *flp-11(syb1445[flp-11-SL2-unc-58(L428F)-linker-mKate2] syb4416[flp-11-deletion]) X*. Generated for this study by outcrossing PHX4416 with N2.

HBR2744 *goeIs3(pmyo-3p::SL1-GCamP3.35-SL2::unc54-3'utr, unc-119(+)); flp-11(syb2193[flp-11-SL2-mKate2-linker-twk-18(e1913)])* X. Generated for this study by crossing PHX2193 and HBR4.

HBR2748 *goeIs3(pmyo-3p::SL1-GCamP3.35-SL2::unc54-3'utr, unc-119(+)); flp-11(syb1445[flp-11-SL2-unc-58(L428F)-linker-mKate2])* X. Generated for this study by crossing HBR2340 and HBR4.

N2 wild type (Bristol). [6]

OH15265 *otIs672(rab-3p::NLS::GCaMP6s, arrd-4p:NLS::GCaMP6s).* [7]

PHX1433 *flp-11(syb1433[flp-11-SL2-egl-23cDNA(A383V)-linker-mKate2])* X*.* Generated by Sunybiotech according to our design for this study.

PHX1445 *aptf-1(gk794) II; flp-11(syb1445)[flp-11-SL2-unc-58(L428F)-linker-mKate2]* X*.* Generated by Sunybiotech according to our design for this study.

PHX1464 *flp-11(syb1464[flp-11-SL2-egl-23cDNA(L229N)-linker-mKate2])* X. Generated by Sunybiotech according to our design for this study.

PHX2193 *flp-11(syb2193[flp-11-SL2-mKate2-linker-twk-18(e1913)])* X*.*

Generated by Sunybiotech according to our design for this study.

PHX2493 *lgc-38(syb2346[flp-11p::dpy-10 site::flp-11 3’UTR], syb2493[ReaChR-linker-mKate2])* III. Generated by Sunybiotech according to our design for this study.

PHX3190 *lgc-38(syb2346[flp-11p::dpy-10 site::flp-11 3’UTR], syb3190[unc-58(e665)-linker(GSGSGSGSG)-mKate2 ])* III. Generated by Sunybiotech according to our design for this study.

PHX4110 *lgc-38(syb2346[flp-11p::dpy-10 site::flp-11 3’UTR], syb4110[unc-58gf-CAI-1.0-linker(GSGSGSGSG)-mKate2]) III.* Generated by Sunybiotech according to our design for this study.

PHX4416 *aptf-1(gk794) II; flp-11(syb1445 syb4416) X.* Generated by Sunybiotech according to our design for this study.

Strain list for this study.

**References**

1. Schwarz J, Spies JP, Bringmann H. Reduced muscle contraction and a relaxed posture during sleep-like Lethargus. Worm. 2012;1(1):12-4. Epub 2012/01/01. doi: 10.4161/worm.19499. PubMed PMID: 24058817; PubMed Central PMCID: PMC3670164.

2. Turek M, Besseling J, Spies JP, Konig S, Bringmann H. Sleep-active neuron specification and sleep induction require FLP-11 neuropeptides to systemically induce sleep. eLife. 2016;5. Epub 2016/03/08. doi: 10.7554/eLife.12499. PubMed PMID: 26949257; PubMed Central PMCID: PMC4805538.

3. Maluck E, Busack I, Besseling J, Masurat F, Turek M, Busch KE, et al. A wake-active locomotion circuit depolarizes a sleep-active neuron to switch on sleep. PLoS biology. 2020;18(2):e3000361. Epub 2020/02/23. doi: 10.1371/journal.pbio.3000361. PubMed PMID: 32078631.

4. Wu Y, Masurat F, Preis J, Bringmann H. Sleep Counteracts Aging Phenotypes to Survive Starvation-Induced Developmental Arrest in C. elegans. Curr Biol. 2018;28(22):3610-24 e8. Epub 2018/11/13. doi: 10.1016/j.cub.2018.10.009. PubMed PMID: 30416057; PubMed Central PMCID: PMCPMC6264389.

5. Koutsoumparis A, Welp LM, Wulf A, Urlaub H, Meierhofer D, Börno S, et al. Sleep neuron depolarization promotes protective gene expression changes and FOXO activation. Current Biology. 2022. doi: <https://doi.org/10.1016/j.cub.2022.04.012>.

6. Brenner S. The genetics of Caenorhabditis elegans. Genetics. 1974;77(1):71-94. Epub 1974/05/01. PubMed PMID: 4366476.

7. Yemini E, Lin A, Nejatbakhsh A, Varol E, Sun R, Mena GE, et al. NeuroPAL: A Multicolor Atlas for Whole-Brain Neuronal Identification in C. elegans. Cell. 2021;184(1):272-88 e11. Epub 2020/12/31. doi: 10.1016/j.cell.2020.12.012. PubMed PMID: 33378642.
