# Supplementary material for: A sleep-active neuron can promote survival while sleep behavior is disturbed
Source: PLoS Genet. 2023 Mar 14;19(3):e1010665. doi: 10.1371/journal.pgen.1010665 (PMC10038310; doi:10.1371/journal.pgen.1010665)
Supplement: S3 Table — (DOCX) [file pgen.1010665.s014.docx]

*aptf-1(gk794)*

CGACAATCTTCCCAAAGACC

CGGATCGATTGCTAGAGAGG

GCTTGGACGGCTTTAGTTGA

*flp-11(syb1445)* these primers were utilized for all strains in which a tool was knocked into the endogenous locus of *flp-11* except for PHX4416

ACGAGGAAGACTTTGCTCCA

AAACTCGCAAAAACGAGGAA

GACACCAATCAAATTCTAGACAGC

*flp-11p::SL2::unc-58(L428F)*

GACCACATGCACGACCTTTT

ATGACTTTCTCCTGCCGTGA

*flp-11(syb4416)*

ACTAGAACAAGCGTCCTCAA

TCCAATTAACACTGACACCA

*lgc-38(syb2346)* these primers were utilized for all strains in which a tool was integrated into the ski-lodge site on chromosome 3.

ATGGCGATGTCATTTTCATGTT

AGACCACCTACCGTTCCAAG

ATCCCAGTTGTTTGACGGTT

*flp-11(tm2706)*

TCTTCCAAATCGAACCAAGG

TAGCCGCTCGTCTCACTTTT

ATGATGAATTCGCCTCAGGA

List of primers utilized in this study.
