# Supplementary material for: A sleep-active neuron can promote survival while sleep behavior is disturbed
Source: PLoS Genet. 2023 Mar 14;19(3):e1010665. doi: 10.1371/journal.pgen.1010665 (PMC10038310; doi:10.1371/journal.pgen.1010665)
Supplement: S4 Table. P — (DOCX) [file pgen.1010665.s015.docx]

| Figure | group 1 | group 2 | statistical test | p-value |
| --- | --- | --- | --- | --- |
| 1F | *RIS::unc-58gf(weak)* | *RIS::unc-58gf(strong)* | Welch test | 2.93904E-5 |
| 2B | wild type | *RIS::twk-18* | Fisher’s Exact test with FDR correction for multiple testing | 2.6472e-26 |
|  | wild type | *RIS::egl23(strong)* | Fisher’s Exact test with FDR correction for multiple testing | 2.0137e-33 |
|  | wild type | *RIS::egl-23(weak)* | Fisher’s Exact test with FDR correction for multiple testing | 0.060809 |
|  | wild type | *RIS::unc-58gf(weak)* | Fisher’s Exact test with FDR correction for multiple testing | 0.23821 |
|  | wild type | *RIS::unc-58gf(strong)* | Fisher’s Exact test with FDR correction for multiple testing | 3.856e-15 |
| 2C | wild type | *RIS::twk-18* | Fisher’s Exact test with FDR correction for multiple testing | 2.9467e-27 |
|  | wild type | *RIS::egl23(strong)* | Fisher’s Exact test with FDR correction for multiple testing | 9.507e-39 |
|  | wild type | *RIS::egl-23(weak)* | Fisher’s Exact test with FDR correction for multiple testing | 1 |
|  | wild type | *RIS::unc-58gf(weak)* | Fisher’s Exact test with FDR correction for multiple testing | 0.011154 |
|  | wild type | *RIS::unc-58gf(strong)* | Fisher’s Exact test with FDR correction for multiple testing | 0.0023327 |
| 3A | wild type | *RIS::twk-18* | Welch test with FDR correction for multiple testing | 0.005595 |
|  | wild type | *RIS::egl23(strong)* | Welch test with FDR correction for multiple testing | 5.6391e-05 |
|  | wild type | *RIS::egl-23(weak)* | Welch test with FDR correction for multiple testing | 0.55774 |
|  | wild type | *RIS::unc-58gf(weak)* | Welch test with FDR correction for multiple testing | 1.2537e-13 |
|  | wild type | *RIS::unc-58gf(strong)* | Welch test with FDR correction for multiple testing | 6.23e-14 |
|  | *RIS::unc-58gf(weak)* | *RIS::unc-58gf(strong)* | Welch test with FDR correction for multiple testing | 0.011028 |
| 4G | wild type pre bout RIS activity | wild type during bout RIS activity | Wilcoxon signed rank test | 8.6542e-12 |
|  | *RIS::egl-23(weak)* pre bout RIS activity | *RIS::egl-23(weak)* during bout RIS activity | Wilcoxon signed rank test | 0.002747 |
|  | *RIS::unc58gf(weak)* pre bout RIS activity | *RIS::unc58gf(weak)* during bout RIS activity | Wilcoxon signed rank test | 1.1124e-15 |
|  | *RIS::unc58gf(strong)* pre bout RIS activity | *RIS::unc58gf(strong)* during bout RIS activity | Wilcoxon signed rank test | 0.7334 |
|  | *RIS::twk-18gf* pre bout RIS activity | *RIS::twk-18gf* during bout RIS activity | Wilcoxon signed rank test | 0.46484 |
|  | wild type pre bout speed | wild type during speed | Wilcoxon signed rank test | 1.4524e-17 |
|  | *RIS::egl-23(weak)* pre bout speed | *RIS::egl-23(weak)* during bout speed | Wilcoxon signed rank test | 3.4946e-18 |
|  | *RIS::unc58gf(weak)* pre bout speed | *RIS::unc58gf(weak)* during bout speed | Wilcoxon signed rank test | 1.1813e-17 |
|  | *RIS::unc58gf(strong)* pre bout speed | *RIS::unc58gf(strong)* during bout speed | Wilcoxon signed rank test | 0.00683 |
|  | *RIS::twk-18gf* pre bout speed | *RIS::twk-18gf* during bout speed | Wilcoxon signed rank test | 0.026855 |
| 4H | wild type | *RIS::twk-18* | Welch test with FDR correction for multiple testing | 4.9986e-09 |
|  | wild type | *RIS::egl23(strong)* | Welch test with FDR correction for multiple testing | 4.9986e-09 |
|  | wild type | *RIS::egl-23(weak)* | Welch test with FDR correction for multiple testing | 0.00539 |
|  | wild type | *RIS::unc-58gf(weak)* | Welch test with FDR correction for multiple testing | 0.00090302 |
|  | wild type | *RIS::unc-58gf(strong)* | Welch test with FDR correction for multiple testing | 5.8071e-09 |
|  | *RIS::unc-58gf(weak)* | *RIS::unc-58gf(strong)* | Welch test with FDR correction for multiple testing | 1.8193e-11 |
| 5G | wild type | *RIS::twk-18* | Welch test with FDR correction for multiple testing | 0.00010291 |
|  | wild type | *RIS::egl23(strong)* | Welch test with FDR correction for multiple testing | 0.0001291 |
|  | wild type | *RIS::egl-23(weak)* | Welch test with FDR correction for multiple testing | 0.00754 |
|  | wild type | *RIS::unc-58gf(weak)* | Welch test with FDR correction for multiple testing | 0.00010291 |
|  | wild type | *RIS::unc-58gf(strong)* | Welch test with FDR correction for multiple testing | 0.0002277 |
| 5H | wild type | *RIS::unc-58gf(strong)* | Welch test | 6.02442e-04 |
| 6B | wild type pre bout neuronal activity | wild type during bout neuronal activity | Wilcoxon signed rank test | 2.3261e-09 |
|  | *RIS::egl-23(weak)* pre bout neuronal activity | *RIS::egl-23(weak)* during bout neuronal activity | Wilcoxon signed rank test | 1.7486e-10 |
|  | *RIS::unc58gf(weak)* pre bout neuronal activity | *RIS::unc58gf(weak)* during bout neuronal activity | Wilcoxon signed rank test | 0.012606 |
|  | *RIS::twk-18gf* pre bout neuronal activity | *RIS::twk-18gf* during bout neuronal activity | Wilcoxon signed rank test | 0.26272 |
|  | *RIS::unc58gf(strong)* pre bout neuronal activity | *RIS::unc58gf(strong)* during bout neuronal activity | Wilcoxon signed rank test | 0.049438 |
|  | wild type pre bout speed | wild type during speed | Wilcoxon signed rank test | 2.8687e-17 |
|  | *RIS::egl-23(weak)* pre bout speed | *RIS::egl-23(weak)* during bout speed | Wilcoxon signed rank test | 8.84e-17 |
|  | *RIS::unc58gf(weak)* pre bout speed | *RIS::unc58gf(weak)* during bout speed | Wilcoxon signed rank test | 3.4075e-15 |
|  | *RIS::twk-18gf* pre bout speed | *RIS:: twk-18gf* during bout speed | Wilcoxon signed rank test | 0.007 |
|  | *RIS::unc58gf(strong)* pre bout speed | *RIS::unc58gf(strong)* during speed | Wilcoxon signed rank test | 0.001709 |
| 6D | wild type | *RIS::twk-18* | Welch test with FDR correction for multiple testing | 6.6283e-05 |
|  | wild type | *RIS::egl23(strong)* | Welch test with FDR correction for multiple testing | 0.025867 |
|  | wild type | *RIS::egl-23(weak)* | Welch test with FDR correction for multiple testing | 0.34555 |
|  | wild type | *RIS::unc-58gf(weak)* | Welch test with FDR correction for multiple testing | 0.38777 |
|  | wild type | *RIS::unc-58gf(strong)* | Welch test with FDR correction for multiple testing | 0.0042 |
| 6F | wild type pre bout RIM activity | wild type during bout RIM activity | Wilcoxon signed rank test | 1.1401e-05 |
|  | *RIS::egl-23(weak)* pre bout RIM activity | *RIS::egl-23(weak)* during bout RIM activity | Wilcoxon signed rank test | 0.03467 |
|  | *RIS::unc58gf(weak)* pre bout RIM activity | *RIS::unc58gf(weak)* during bout RIM activity | Wilcoxon signed rank test | 0.001616 |
|  | *RIS::twk-18gf* pre bout RIM activity | *RIS::twk-18gf* during bout RIM activity | Wilcoxon signed rank test | 0.30078 |
|  | *RIS::unc58gf(strong)* pre bout RIM activity | *RIS::unc58gf(strong)* during bout RIM activity | Wilcoxon signed rank test | 0.1288 |
|  | wild type pre bout speed | wild type during speed | Wilcoxon signed rank test | 4.3681e-18 |
|  | *RIS::egl-23(weak)* pre bout speed | *RIS::egl-23(weak)* during bout speed | Wilcoxon signed rank test | 2.3544e-13 |
|  | *RIS::unc58gf(weak)* pre bout speed | *RIS::unc58gf(weak)* during bout speed | Wilcoxon signed rank test | 1.0993e-12 |
|  | *RIS::twk-18gf* pre bout speed | *RIS::twk-18gf* during bout speed | Wilcoxon signed rank test | 2.732e-05 |
|  | *RIS::unc58gf(strong)* pre bout speed | *RIS::unc58gf(strong)* during bout speed | Wilcoxon signed rank test | 1.1004e-07 |
| 6H | wild type | *RIS::twk-18* | Welch test with FDR correction for multiple testing | 0.00055411 |
|  | wild type | *RIS::egl23(strong)* | Welch test with FDR correction for multiple testing | 0.00735 |
|  | wild type | *RIS::egl-23(weak)* | Welch test with FDR correction for multiple testing | 0.00055411 |
|  | wild type | *RIS::unc-58gf(weak)* | Welch test with FDR correction for multiple testing | 0.00645 |
|  | wild type | *RIS::unc-58gf(strong)* | Welch test with FDR correction for multiple testing | 8.7669e-06 |
| 7D | wild type | *RIS::twk-18* | Welch test | 7.04184e-04 |
|  | wild type | *RIS::unc-58gf(strong)* | Welch test | 0.0183 |
| 7E | wild type | *RIS::twk-18* | Welch test | 0.00183 |
|  | wild type | *RIS::unc-58gf(strong)* | Welch test | 0.00398 |
| 7F | wild type quiescent bouts | *RIS::twk-18* quiescent bouts | Welch test | 0.03172 |
|  | wild type quiescent bouts | *RIS::unc-58gf(strong)* quiescent bouts | Welch test | 0.54995 |
|  | wild type mobile bouts | *RIS::twk-18* mobile bouts | Welch test | 0.00283 |
|  | wild type mobile bouts | *RIS::unc-58gf(strong)* mobile bouts | Welch test | 0.84769 |
|  | wild type quiescent bouts | wild type mobile bouts | Wilcoxon signed rank test | 0.00103 |
|  | *RIS::twk-18* quiescent bouts | *RIS::twk-18* mobile bouts | Wilcoxon signed rank test | 2.26067e-07 |
|  | *RIS::unc-58gf(strong)* quiescent bouts | *RIS::unc-58gf(strong)* mobile bouts | Wilcoxon signed rank test | 0.00103 |
| 8A | wild type quiescent bouts | *RIS::twk-18* quiescent bouts | Welch test | 0.00409 |
|  | wild type quiescent bouts | *RIS::unc-58gf(strong)* quiescent bouts | Welch test | 0.04721 |
|  | wild type mobile bouts | *RIS::twk-18* mobile bouts | Welch test | 0.06568 |
|  | wild type mobile bouts | *RIS::unc-58gf(strong)* mobile bouts | Welch test | 0.00162 |
|  | wild type quiescent bouts | wild type mobile bouts | Wilcoxon signed rank test | 7.45058e-09 |
|  | *RIS::twk-18* quiescent bouts | *RIS::twk-18* mobile bouts | Wilcoxon signed rank test | 0.00781 |
|  | *RIS::unc-58gf(strong)* quiescent bouts | *RIS::unc-58gf(strong)* mobile bouts | Wilcoxon signed rank test | 1.90735e-06 |
| 8B | wild type quiescent bouts | *RIS::twk-18* quiescent bouts | Welch test | 5.57801e-10 |
|  | wild type quiescent bouts | *RIS::unc-58gf(strong)* quiescent bouts | Welch test | 0.22981 |
|  | wild type mobile bouts | *RIS::twk-18* mobile bouts | Welch test | 0.74543 |
|  | wild type mobile bouts | *RIS::unc-58gf(strong)* mobile bouts | Welch test | 0.22981 |
|  | wild type quiescent bouts | wild type mobile bouts | Wilcoxon signed rank test | 9.56917e-05 |
|  | *RIS::twk-18* quiescent bouts | *RIS::twk-18* mobile bouts | Wilcoxon signed rank test | 2.88885e-05 |
|  | *RIS::unc-58gf(strong)* quiescent bouts | *RIS::unc-58gf(strong)* mobile bouts | Wilcoxon signed rank test | 0.00166 |
| 8F | wild type quiescent bouts | *RIS::twk-18* quiescent bouts | Welch test | 0.71373 |
|  | wild type quiescent bouts | *RIS::unc-58gf(strong)* quiescent bouts | Welch test | 0.00957 |
|  | wild type mobile bouts | *RIS::twk-18* mobile bouts | Welch test | 0.76635 |
|  | wild type mobile bouts | *RIS::unc-58gf(strong)* mobile bouts | Welch test | 0.03191 |
|  | wild type quiescent bouts | wild type mobile bouts | Wilcoxon signed rank test | 1.29926e-04 |
|  | *RIS::twk-18* quiescent bouts | *RIS::twk-18* mobile bouts | Wilcoxon signed rank test | 0.01563 |
|  | *RIS::unc-58gf(strong)* quiescent bouts | *RIS::unc-58gf(strong)* mobile bouts | Wilcoxon signed rank test | 1.49012e-08 |
| 8G | wild type pre bout muscle activity | wild type during bout muscle activity |  | 2.2874e-10 |
|  | *RIS::twk-18gf* pre bout muscle activity | *RIS::twk-18gf* during bout muscle activity |  | 0.82031 |
|  | *RIS::unc58gf(strong)* pre bout muscle activity | *RIS::unc58gf(strong)* during bout muscle activity |  | 1.6621e-07 |
|  | wild type pre bout speed | wild type during speed |  | 1.3599e-05 |
|  | *RIS::twk-18gf* pre bout speed | *RIS::twk-18gf* during bout speed |  | 9.9904e-06 |
|  | *RIS::unc58gf(strong)* pre bout speed | *RIS::unc58gf(strong)* during bout speed |  | 6.7468e-05 |
| 8H | wild type | *RIS::twk-18gf* |  | 0.01585 |
|  | wild type | *RIS::unc-58gf(strong)* |  | 0.02974 |
| 9A | wild type pre stimulation RIS activity | wild type during stimulation RIS activity | Wilcoxon signed rank test | 0.00012207 |
|  | wild type pre stimulation speed | wild type during stimulation speed | Wilcoxon signed rank test | 0.001709 |
|  | *RIS::unc-58gf(strong)* pre stimulation RIS activity | *RIS::unc-58gf(strong)* during stimulation RIS activity | Wilcoxon signed rank test | 0.39443 |
|  | *RIS::unc-58gf(strong)* pre stimulation speed | *RIS::unc-58gf(strong)* during stimulation speed | Wilcoxon signed rank test | 0.97574 |
| 9B | wild type pre stimulation RIS activity | wild type during stimulation RIS activity | Wilcoxon signed rank test | 0.54688 |
|  | wild type pre stimulation speed | wild type during stimulation speed | Wilcoxon signed rank test | 0.38281 |
|  | *RIS::unc-58gf(strong)* pre stimulation RIS activity | *RIS::unc-58gf(strong)* during stimulation RIS activity | Wilcoxon signed rank test | 0.17881 |
|  | *RIS::unc-58gf(strong)* pre stimulation speed | *RIS::unc-58gf(strong)* during stimulation speed | Wilcoxon signed rank test | 0.60509 |
| 10C | *RIS::ReaChR(-ATR)* first hour GCaMP | *RIS::ReaChR(+ATR)* first hour GCaMP | Welch test | 0.03655 |
|  | *RIS::ReaChR(-ATR)* last hour GCaMP | *RIS::ReaChR(+ATR)* last hour GCaMP | Welch test | 0.049849 |
|  | *RIS::ReaChR(-ATR)* first hour speed | *RIS::ReaChR(+ATR)* first hour speed | Welch test | 0.0005971 |
| 10E | *RIS::ReaChR(-ATR)* | *RIS::ReaChR(+ATR)* | Welch test | 0.02344 |
| 10F | *RIS::ReaChR(-ATR)* | *RIS::ReaChR(+ATR)* | Welch test | 0.00584 |
| 10G | *RIS::ReaChR(+ATR)* | *Wild type (+ATR)* | Logrank test | 0.09 |
|  |  |  | Fisher’s Exact test | 0.02 |
|  | *RIS::ReaChR(-ATR)* | *Wild type (-ATR)* | Logrank test | 0.82 |
| 11C | Wild type control | Wild type stimulation | Welch test | 1.94557e-11 |
|  | Wild type stimulation | *RIS::twk-18gf* stimulation | Welch test | 5.80697e-05 |
| 11D | Wild type stimulation | *RIS::twk-18gf* stimulation | Welch test | 0.01972 |
| 12A | wild type | *flp-11(-)* | Welch test with FDR correction for multiple testing | 4.013e-14 |
|  | wild type | *RIS::unc-58gf(strong)* | Welch test with FDR correction for multiple testing | 4.0915e-15 |
|  | wild type | *RIS::unc-58gf(strong), flp-11(-)* | Welch test with FDR correction for multiple testing | 3.6044e-14 |
| 12B | wild type | *flp-11(-)* | Fisher’s Exact test with FDR correction for multiple testing | 1.05E-22 |
|  | wild type | *RIS::unc-58gf(strong)* | Fisher’s Exact test with FDR correction for multiple testing | 0.00016 |
|  | wild type | *RIS::unc-58gf(strong), flp-11(-)* | Fisher’s Exact test with FDR correction for multiple testing | 9.52E-14 |
|  | *flp-11(-)* | *RIS::unc-58gf(strong), flp-11(-)* | Fisher’s Exact test with FDR correction for multiple testing | 6.36E-05 |
|  | *RIS::unc-58gf(strong)* | *RIS::unc-58gf(strong), flp-11(-)* | Fisher’s Exact test with FDR correction for multiple testing | 5.33E-22 |
| S2A | wild type | *RIS::unc-58gf(strong)* | Fisher’s Exact test | 7.26E-08 |
|  | wild type | *RIS::unc-58gf(strong), aptf-1(-)* | Fisher’s Exact test | 1.4798e-19 |
|  | *RIS::unc-58gf(strong)* | *RIS::unc-58gf(strong), aptf-1(-)* | Fisher’s Exact test | 3.5813e-16 |
|  | *aptf-1(-)* | *RIS::unc-58gf(strong), aptf-1(-)* | Fisher’s Exact test | 0.0026726 |
| S2B | wild type | *RIS::unc-58gf(strong)* | Welch test | 8.05065E-4 |
|  | wild type | *aptf-1(-)* | Welch test | 2.60456E-13 |
|  | wild type | *RIS::unc-58gf(strong), aptf-1(-)* | Welch test | 8.89446E-16 |
|  | *aptf-1(-)* | *RIS::unc-58gf(strong), aptf-1(-)* | Welch test | 0.0767 |
|  | *RIS::unc-58gf(strong)* | *RIS::unc-58gf(strong), aptf-1(-)* | Welch test | 0.00447 |
| S3A | wild type | *RIS::twk-18* | Welch test with FDR correction for multiple testing | 2.9535e-08 |
|  | wild type | *RIS::egl23(strong)* | Welch test with FDR correction for multiple testing | 3.1271e-13 |
|  | wild type | *RIS::egl-23(weak)* | Welch test with FDR correction for multiple testing | 0.44566 |
|  | wild type | *RIS::unc-58gf(weak)* | Welch test with FDR correction for multiple testing | 8.7484e-24 |
|  | wild type | *RIS::unc-58gf(strong)* | Welch test with FDR correction for multiple testing | 3.4427e-20 |
|  | *RIS::unc-58gf(weak)* | *RIS::unc-58gf(strong)* | Welch test with FDR correction for multiple testing | 0.96149 |
| S3B | wild type | *RIS::twk-18* | Welch test with FDR correction for multiple testing | 1.4404e-05 |
|  | wild type | *RIS::egl23(strong)* | Welch test with FDR correction for multiple testing | 1.3078e-05 |
|  | wild type | *RIS::egl-23(weak)* | Welch test with FDR correction for multiple testing | 0.98035 |
|  | wild type | *RIS::unc-58gf(weak)* | Welch test with FDR correction for multiple testing | 0.80731 |
|  | wild type | *RIS::unc-58gf(strong)* | Welch test with FDR correction for multiple testing | 0.0018333 |
| S4 | wild type | *RIS::unc-58gf(strong)* | Welch test | 0.44996 |
| S8 | wild type | *RIS::twk-18* | Welch test with FDR correction for multiple testing | 0.05085 |
|  | wild type | *RIS::egl23(strong)* | Welch test with FDR correction for multiple testing | 0.10085 |
|  | wild type | *RIS::egl-23(weak)* | Welch test with FDR correction for multiple testing | 0.41311 |
|  | wild type | *RIS::unc-58gf(weak)* | Welch test with FDR correction for multiple testing | 0.16855 |
|  | wild type | *RIS::unc-58gf(strong)* | Welch test with FDR correction for multiple testing | 0.79677 |
| S9B | wild type | *flp-11(-)* | Fisher’s Exact test with FDR correction for multiple testing | 3.36E-24 |
|  | wild type | *RIS::unc-58gf(strong)* | Fisher’s Exact test with FDR correction for multiple testing | 0.081516 |
|  | wild type | *RIS::unc-58gf(strong), flp-11(-)* | Fisher’s Exact test with FDR correction for multiple testing | 6.83E-18 |
|  | *flp-11(-)* | *RIS::unc-58gf(strong), flp-11(-)* | Fisher’s Exact test with FDR correction for multiple testing | 0.081516 |
|  | *RIS::unc-58gf(strong)* | *RIS::unc-58gf(strong), flp-11(-)* | Fisher’s Exact test with FDR correction for multiple testing | 3.00E-13 |
| S10 | Wild type | *RIS::unc-58gf(strong)* | Welch test | 9.106e-13 |
|  | Wild type | DAF-16(-) | Welch test | 4.73842e-08 |
|  | Wild type | *RIS::unc-58gf(strong);* DAF-16(-) | Welch test | 1.28118e-12 |
|  | *RIS::unc-58gf(strong)* | *RIS::unc-58gf(strong);* DAF-16(-) | Welch test | 0.31948 |
|  | DAF-16(-) | *RIS::unc-58gf(strong);* DAF-16(-) | Welch test | 2.86134e-04 |

p-values and statistical tests for all experiments
